# Regulation of Miwi-mediated mRNA stabilization by Ck137956/Tssa is essential for male fertility

**DOI:** 10.1186/s12915-023-01589-z

**Published:** 2023-04-17

**Authors:** Yu Chen, Xiangzheng Zhang, Jiayin Jiang, Mengjiao Luo, Haixia Tu, Chen Xu, Huanhuan Tan, Xin Zhou, Hong Chen, Xudong Han, Qiuling Yue, Yueshuai Guo, Ke Zheng, Yaling Qi, Chenghao Situ, Yiqiang Cui, Xuejiang Guo

**Affiliations:** 1 State Key Laboratory of Reproductive Medicine and Offspring Health, Nanjing Medical University, Nanjing, 211166 China; 2 School of Medicine, Southeast University, Nanjing, 210009 China

**Keywords:** Ck137956/Tssa, Miwi, mRNA stability, Spermiogenesis, Male infertility

## Abstract

**Background:**

Sperm is formed through spermiogenesis, a highly complex process involving chromatin condensation that results in cessation of transcription. mRNAs required for spermiogenesis are transcribed at earlier stages and translated in a delayed fashion during spermatid formation. However, it remains unknown that how these repressed mRNAs are stabilized.

**Results:**

Here we report a Miwi-interacting testis-specific and spermiogenic arrest protein, Ck137956, which we rename Tssa. Deletion of Tssa led to male sterility and absence of sperm formation. The spermiogenesis arrested at the round spermatid stage and numerous spermiogenic mRNAs were down-regulated in *Tssa*^*−/−*^ mice. Deletion of Tssa disrupted the localization of Miwi to chromatoid body, a specialized assembly of cytoplasmic messenger ribonucleoproteins (mRNPs) foci present in germ cells. We found that Tssa interacted with Miwi in repressed mRNPs and stabilized Miwi-interacting spermiogenesis-essential mRNAs.

**Conclusions:**

Our findings indicate that Tssa is indispensable in male fertility and has critical roles in post-transcriptional regulations by interacting with Miwi during spermiogenesis.

**Supplementary Information:**

The online version contains supplementary material available at 10.1186/s12915-023-01589-z.

## Background

Spermatogenesis is a complex and highly regulated process which contains mitotic cell division, meiosis and spermiogenesis [1, 2]. The process requires coordinated interactions between multiple molecules whose expression is precisely controlled in time and space. During spermiogenesis, haploid round spermatids transform into specialized and uniquely shaped elongated spermatids with the gradual condensation of the nuclei [3]. The transcription cessation in condensing elongating spermatids results in the temporal uncoupling between mRNA transcription and translation. Therefore, the long-term mRNA storage in free messenger ribonucleoproteins (mRNPs) is needed to provide necessary mRNAs for later protein synthesis in transcriptionally inactive cells [4]. For example, spermatids-specific mRNAs protamine 1 (Prm1) and protamine 2 (Prm2) are repressed translationally in round spermatids and actively translated in elongated spermatids [5]. Premature translation of Prm1 mRNA causes precocious condensation of spermatid nuclear DNA, abnormal head morphogenesis and spermatid differentiation arrest in mice [6], indicating the importance of uncoupling of transcription and translation during spermatid development. Thus, post-transcriptional regulation of mRNA stability and translation is important for male germ cell development [7, 8].

The Piwi proteins are a subfamily of the Ago/Piwi proteins that are predominantly present in animal germline [9]. Miwi, a cytoplasmic Piwi protein binding to 29 ~ 31nt Piwi-interacting RNAs (piRNAs), is expressed from pachytene spermatocytes to round spermatids [10]. Deletion of Miwi causes an arrest at the round spermatid stage of spermatogenesis. Miwi/piRNAs play critical roles in germ cell development, including suppressing transposons, eliminating mRNAs and activating translation [11–15]. Besides, without piRNA guides, Miwi also binds directly to spermatogenic mRNAs and participates in the formation of the repressive mRNPs in post-meiotic spermatids [16]. The absence of Miwi leads to decreased spermiogenic mRNAs stored in mRNPs. However, the molecular mechanisms involved in the stability regulation of these spermiogenic mRNAs during spermatid differentiation are still not well known.

In our previous report [17], we quantified protein expression levels of testis-specific genes in different stages of germ cell development using LC–MS/MS, and identified a testis-specific uncharacterized protein, Ck137956, which was further found to interact with Miwi. It is mainly expressed in pachytene spermatocytes and round spermatids. *Ck137956* deficiency in mice caused complete male infertility, no sperm production and arrest at the round spermatid stage. Deletion of Ck137956 led to disrupted chromatoid body localization of Miwi and decreased Miwi protein expression. As a cytoplasmic protein, Ck137956 interacts with Miwi, participates in the formation of mRNPs, and stabilizes repressed spermiogenic mRNAs without affecting their transcription. We provide insights into the regulatory mechanism of Ck137956 in Miwi-stabilized mRNAs in mRNPs during spermiogenesis.

## Results

### Expression of Ck137956 in mouse tissues and testis development

**Fig. 1:**
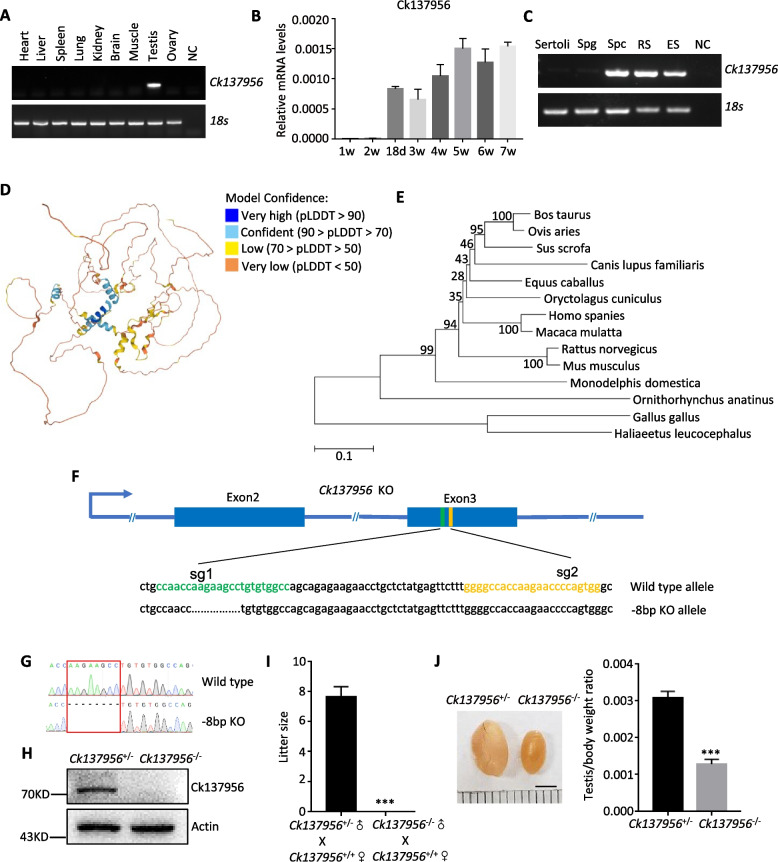
Expression of Ck137956 and fertility of Ck137956 knockout mice. **A** Ck137956 mRNA expression was detected in various tissues from adult mice by RT-PCR with 18S as a loading control. NC, negative control. **B** qRT-PCR analysis of Ck137956 expression in postnatal mouse testes. *n* = 3. Data were presented as the mean ± SD. **C** The presence of Ck137956 mRNA was evaluated in different types of spermatogenic cells with 18S as a loading control. Spg, spermatogonia; Spc, pachytene spermatocytes; RS, round spermatids; ES, elongated spermatids. **D** Structure of mouse Ck137956 protein predicted by AlphaFold. **E** Phylogenetic tree showing the evolutionary distance of Ck137956 among different animals with branch lengths in units representing the number of amino acids substituted per site. The numbers exhibit bootstrap values. **F** Schematic diagram of knockout strategy by CRISPR/Cas9. Yellow and green: the single guide RNA (sgRNA) targeting sites. **G** Sanger sequencing showing eight bases deletion of the *Ck137956* gene. **H** Expression of Ck137956 protein in *Ck137956*^+/-^ and *Ck137956*^*−/−*^ testis with Actin as a loading control. **I** Litter size of *Ck137956*^+/-^ and *Ck137956*^*−/−*^ male mice mated with wildtype female mice. *n* = 6. Data were presented as the mean ± SEM. ***p < 0.001. **J** Morphology of adult *Ck137956*^+/-^ and *Ck137956*^*−/−*^ testes and statistics of testis/body weight ratio. Scale bar: 3 mm. *n* = 6. Data were presented as the mean ± SEM. ***p < 0.001

To analyze the tissue distribution of Ck137956, we performed reverse transcription polymerase chain reaction (RT-PCR) analysis. We found that Ck137956 mRNA was specifically expressed in testis among the nine adult mouse tissues of heart, liver, spleen, lung, kidney, brain, muscle, testis and ovary (Fig. 1A). The 1-, 2-, 18dpp-, 3-, 4-, and 5- to 7-week postnatal mouse testis coincides with the development of spermatogonia, early pachytene spermatocytes, late pachytene spermatocytes, round spermatids, elongated spermatids, and sperm, respectively. The qRT-PCR analysis showed that Ck137956 started to express in 18dpp testis, indicating its expression in late pachytene spermatocytes (Fig. 1B). And consistently, Ck137956 protein was also expressed from 18-day postnatal testis according to Western blot analysis (Additional file 1: Fig. S1A). Further analysis of its expression in purified mouse Sertoli cells, spermatogonia, pachytene spermatocytes, round spermatids and elongated spermatids showed that Ck137956 was expressed in the pachytene spermatocytes and round spermatids, weakly in the elongated spermatids, but not in Sertoli cells (Fig. 1C).

Ck137956 is an uncharacterized protein. With the development of the neural network-based model, protein structure prediction has been considerably improved. Using AlphaFold, we predicted the Ck137956 protein structure, and found that Ck137956 had disordered domains in its N-terminal (20-40aa) and C-terminal region (541-570aa) (Fig. 1D). To examine the conservation of Ck137956 protein sequence during evolution, we further performed phylogenetic analysis of Ck137956 from the major animal branches (Fig. 1E). The results showed that Ck137956 was present from birds to humans, indicating that Ck137956 was conserved beyond mammals. The evolutionary relationship throughout major lineages of animals suggested that Ck137956 might play important roles in spermatogenesis in most animal species.

### Testis-specific Ck137956 is essential for male fertility and spermatid development in mouse

To study the in vivo functions of Ck137956 in spermatogenesis, we used CRISPR/Cas9 system to generate *Ck137956* knockout (KO) mice (Fig. 1F), and generated 8 bp deletion in *Ck137956* (Fig. 1G). To evaluate the efficiency of *Ck137956* deletion, we performed Western blot analysis to examine the expression level of Ck137956 protein. The results showed the absence of Ck137956 protein in adult *Ck137956*^*−/−*^ mouse testis (Fig. 1H), indicating successful knockout of Ck137956 in spermatogenic cells. In addition, fertility tests were conducted for 3 months, and the results showed that *Ck137956*^*−/−*^ males produced no offspring and were sterile (Fig. 1I). Furthermore, *Ck137956*^*−/−*^ males exhibited reduced testis weight compared to the controls, while the body weight was unaffected (Fig. 1J and Additional file 1: Fig. S1B-C).

**Fig. 2:**
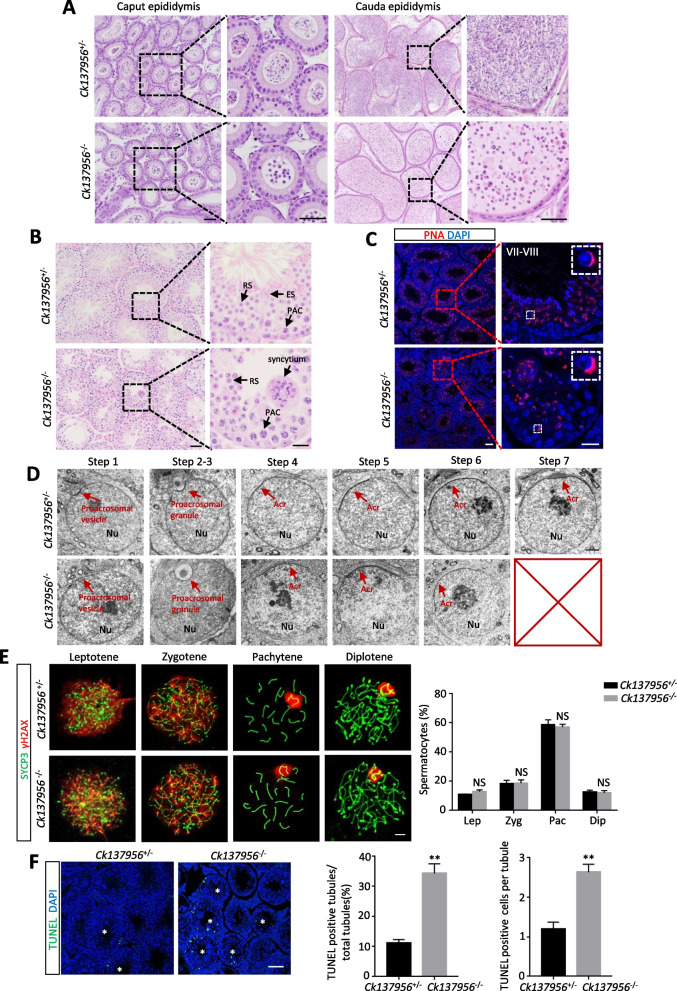
Phenotype analysis of Ck137956 knockout male mice. **A** HE-stained sections of the caput and cauda epididymis from *Ck137956*^+/-^ and *Ck137956*^*−/−*^ mice. Scale bars: 50 μm. **B** HE-stained sections of the testes from *Ck137956*^+/-^ and *Ck137956*^*−/−*^ mice. PAC, pachytene spermatocytes; RS, round spermatids; ES, elongated spermatids. Scale bars: 50 μm. **C** Immunofluorescence analysis of seminiferous tubules from *Ck137956*^+/-^ and *Ck137956*^*−/−*^ testes using PNA (red) stained the acrosome and DAPI (blue) stained the nuclei. Magnification of round spermatids were shown in white. Scale bars: 50 μm. **D** Ultrastructural analysis of *Ck137956*^+/-^ and *Ck137956*^*−/−*^ round spermatids by transmission electron microscopy. The developmental stage of round spermatids was determined by acrosomal angles. Nu, nucleus; Acr, acrosome. Scale bar: 1 μm. **E** Immunofluorescence staining of SYCP3 (green) and γH2AX (red) on spread nuclei of spermatocytes from *Ck137956*^+/-^ and *Ck137956*^*−/−*^ testis. Scale bar: 5 μm. *n* = 3. Data were presented as the mean ± SEM. NS, not significant. **F** TUNEL staining (green) and quantitative analysis of *Ck137956*^+/-^ and *Ck137956*.^*−/−*^ seminiferous tubules. DAPI (blue) stained the nuclei. Asterisks indicated TUNEL-positive tubules. Scale bar: 50 μm. *n* = 3. Data were presented as the mean ± SEM. **p < 0.01

To explore the cause of male infertility in *Ck137956*^*−/−*^ mice, we performed histological analysis of epididymis by HE-staining. We found that sperm were absent in the caput and cauda epididymis, instead the epididymis is filled with round cells (Fig. 2A). As no sperm were observed in the epididymis, we further examined the spermatogenic defects after Ck137956 deletion, and found that *Ck137956*^*−/−*^ testes lacked elongated spermatids and sperm, and contained syncytium with multiple nuclei of round spermatids (Fig. 2B). Peanut agglutinin (PNA) staining was performed to analyze the acrosome angles with developmental steps of round spermatids. Compared to the step7 round spermatids in controls, *Ck137956*^*−/−*^ round spermatids arrested at the step 6 with the acrosome angles of 95–120° (Fig. 2C).

To analyze the ultrastructural changes of round spermatids after deletion of Ck137956, we performed a transmission electron microscopy (TEM) analysis. We found that similar to the controls, *Ck137956*^*−/−*^ round spermatids showed acrosome formation with appearance of proacrosomal vesicle and attachment of flattening of acrosome granule to the nuclear membrane, and formation of nuclear theca. The formation of round spermatids with the acrosome angles of 95–120° indicated the development of step 6 round spermatids. As expected, the step7 round spermatids were not detected, consistent with the results of PNA-staining (Fig. 2D).

Ck137956 is expressed from spermatocytes to spermatids. To evaluate the function of *Ck137956* in meiosis of spermatocytes, we analyzed the meiotic development of spermatocytes by chromosome spread and immunostaining of SYCP3 and γH2AX. The results showed that the *Ck137956*^*−/−*^ spermatocytes displayed a similar distribution of leptotene, zygotene, pachytene and diplotene stages to the *Ck137956*^+/-^ controls (Fig. 2E). Thus, the meiotic progression of spermatocytes was not affected by Ck137956 deletion. Due to the developmental arrest of round spermatids in testis, we further analyzed the apoptotic state of testicular cells by TUNEL staining. We found that both the percentage of TUNEL positive tubules and the average number of TUNEL positive cells per tubule were significantly increased in *Ck137956*^*−/−*^ males compared with the *Ck137956*^+/-^ controls (Fig. 2F). Thus, Ck137956 was indispensable in male fertility and played a critical role in spermatids development.

### Majority of spermiogenesis-related differentially expressed mRNAs are down- regulated after* Ck137956* deletion

**Fig. 3:**
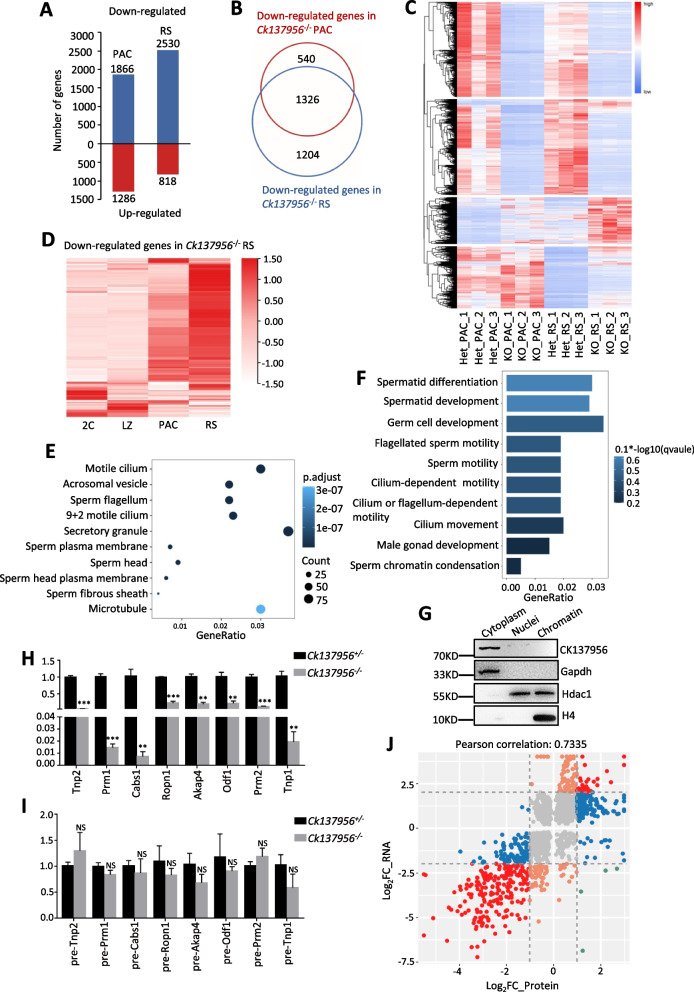
Gene expression analysis of Ck137956 knockout pachytene spermatocytes and round spermatids. **A** Number of differentially expressed genes in *Ck137956*^*−/−*^ pachytene spermatocytes and round spermatids. PAC, pachytene spermatocytes; RS, round spermatids. **B** Venn diagram showing overlap between down-regulated genes in *Ck137956*^*−/−*^ pachytene spermatocytes and those in *Ck137956*^*−/−*^ round spermatids. **C** Heatmap of genes with differential expression (Log_2_FoldChange > 2 and adjusted *P* < 0.01) between *Ck137956*^+/-^ and *Ck137956*^*−/−*^ mice in pachytene spermatocytes or round spermatids. **D** Heatmap of gene expression during spermatogenesis for genes down-regulated in *Ck137956*^*−/−*^ RS. 2C: spermatogonia and somatic cells; LZ: leptotene/zygotene spermatocytes; PAC, pachytene spermatocytes; RS, round spermatids. **E–F** Enriched cellular component terms (**E**) and biological process terms (**F**) in the down-regulated genes in *Ck137956*^−/−^ round spermatids. **G** Western blot analysis of Ck137956 expression in cytoplasm, nuclei, and chromatin. Gapdh, Hdac1 and histone H4 served as cytoplasm, nuclei and chromatin controls, respectively. **H-I** qRT-PCR analysis of the levels of mRNAs (**H**) and pre-mRNAs (**I**) of spermatogenesis-related genes in day-24 *Ck137956*^+/-^ and *Ck137956*^*−/−*^ testes. *n* = 3. Data were presented as the mean ± SEM. NS, not significant, **p < 0.01, ***p < 0.001. **J** Scatterplot of Log_2_FoldChange between transcriptome and proteome after *Ck137956* deletion in round spermatids

Ck137956 is expressed in spermatocytes and spermatids. To characterize the molecular defects in spermatogenesis after Ck137956 deletion, we performed RNA-seq analysis of purified pachytene spermatocytes and round spermatids from *Ck137956*^+/-^ and *Ck137956*^*−/−*^ male testis (Additional file 1: Fig. S2A, Additional files 3 and 4). As a result, 3152 genes exhibited differential expressions in pachytene spermatocytes, among which 1866 (59%) were down-regulated (Log_2_FoldChange < -2, adjusted *p* < 0.01). In round spermatids, 3348 genes were identified to be differentially expressed, and 2530 (76%) were down-regulated (Log_2_FoldChange < -2, adjusted *p* < 0.01) (Fig. 3A). Approximate 40% of the differentially expressed genes exhibited decreased expression both in *Ck137956*^*−/−*^ pachytene spermatocytes and round spermatids and 1326 (71%) down-regulated genes in pachytene spermatocytes were also down-regulated in round spermatids (Fig. 3B). Further heatmap analysis of the differentially expressed genes showed the majority of the differentially expressed genes displayed similar changes of gene expression between pachytene spermatocytes and round spermatids (Fig. 3C).

We performed the gene ontology enrichment analysis for up-regulated proteins in *Ck137956*^*−/−*^ mice. Biological process (BP) term analysis revealed that the proteins up-regulated in pachytene spermatocytes were enriched in “male gonad development” and “fertilization”, while the BP terms in proteins up-regulated in round spermatids were enriched in “cellular modified amino acid metabolic process” and “lipid oxidation” (Additional file 1: Fig. S2B-C). Cellular component (CC) term analysis showed that up-regulated genes in *Ck137956*^*−/−*^ pachytene spermatocytes were significantly enriched in “sperm head”; “acrosome vesicle”; “9 + 2 motile cilium” and “sperm flagellum” (Additional file 1: Fig. S2D), while up-regulated genes in round spermatids showed no enriched term. It seems that up-regulated genes in round spermatids are not enriched in spermiogenesis-related terms. Furthermore, down-regulated genes in *Ck137956*^*−/−*^ pachytene spermatocytes and round spermatids were abundant at the post-meiotic spermatid stage (Additional file 1: Fig. S2E and Fig. 3D), when compared to the transcriptomes of stage-specific spermatogenic cell populations reported previously [18], including spermatogonia/somatic cells, leptotene/zygotene spermatocytes, pachytene spermatocytes and round spermatids. Gene Ontology (GO) analysis of down-regulated genes in pachytene spermatocytes revealed that terms related to “acrosome vesicle”; “sperm head”; “motile cilium”; “sperm flagellum” and “9 + 2 motile cilium” were enriched in cellular component (CC) (Additional file 1: Fig. S2F), while biological processes (BP) showed significant enrichment in the terms related to “spermatid differentiation”; “sperm motility” and “sperm chromatin condensation” (Additional file 1: Fig. S2G). Similarly, down-regulated genes in round spermatids are also enriched in terms related to spermiogenesis, including cellular components “motile cilium”; “acrosomal vesicle”; “sperm flagellum” and “sperm head” (Fig. 3E) and biological processes “spermatid differentiation”; “sperm motility” and “cilium movement” (Fig. 3F), suggesting that these down-regulated genes were highly involved in spermiogenesis.

Altogether, due to the large amounts of spermiogenesis-related mRNAs being down-regulated in *Ck137956*^*−/−*^ spermatids, we speculated that Ck137956 might regulate transcriptional activity or stability of spermatogenic mRNAs. The subcellular localization of a protein affects its function. To examine the subcellular location of Ck137956 in the testis, we isolated cytoplasm, nuclei and chromatin. Western blot results showed that Ck137956 was localized exclusively in the cytoplasm (Fig. 3G), indicating that Ck137956 may not regulate the transcription of the down-regulated genes. To further confirm that the transcription of down-regulated genes in *Ck137956*^*−/−*^ spermatids was not affected, we evaluated the pre-mRNA levels of down-regulated genes essential for spermiogenesis, including Tnp2, Prm1, Cabs1, Ropn1, Akap4, Odf1, Prm2 and Tnp1 [19–25] by qRT-PCR in 24-day *Ck137956*^*−/−*^ testis, which contained similar proportions of testicular cells to the control testis (Additional file 1: Fig. S2H). Although these genes were down-regulated at expression levels in *Ck137956*^*−/−*^ testis by qRT-PCR, consistent with the RNA-seq data (Fig. 3H), their pre-mRNA levels remained unchanged (Fig. 3I). Therefore, their mRNAs may have lower stability and be regulated mainly at the post-transcriptional level in the cytoplasm after Ck137956 deletion. Furthermore, we measured protein expression levels using multiplexed tandem mass tags (TMT) labelling-based tandem mass spectrometry (LC–MS/MS) approach in *Ck137956*^*−/−*^ round spermatids (Additional file 5). Comparative analysis of both transcriptome and proteome levels in *Ck137956*^*−/−*^ round spermatids showed a high correlation between these two levels (Fig. 3J), and proteins down-regulated were mainly caused by lower expressions of their corresponding mRNAs. Thus, Ck137956 mainly affected the stability of mRNAs, which resulted in changes of corresponding proteins.

### Cytoplasmic Ck137956 interacts with and regulates the stability of Miwi

**Fig. 4:**
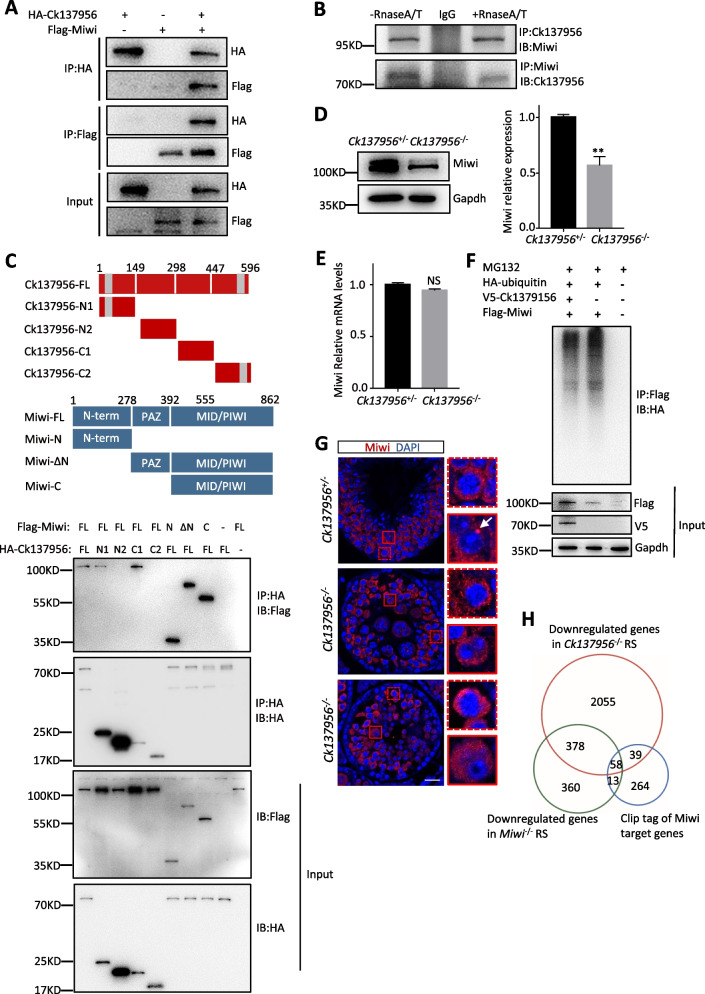
Ck137956 regulates ubiquitination and localization of Miwi. **A** Co-IP assay of interaction between HA-Ck137956 and Flag-Miwi in HEK-293 T cells. **B** Co-IP assay of interaction between Ck137956 and Miwi in testis with or without RnaseA/T treatment. **C** Domain mapping of the Ck137956-Miwi interaction by co-transfection with HA-tagged Ck137956 truncations and Flag-tagged Miwi truncations. The scheme of Ck137956 and Miwi truncations is shown in the upper panel. The grey indicates disordered region. **D** The levels of Miwi protein expression in day-24 *Ck137956*^+/-^ and *Ck137956*^*−/−*^ testis. Tubulin was used as a loading control. *n* = 3. Data were presented as the mean ± SEM. **p < 0.01. **E** mRNA expression levels of Miwi quantified by qRT-PCR in day-24 *Ck137956*^+/-^ and *Ck137956*^*−/−*^ testis. *n* = 3. Data were presented as the mean ± SEM. NS, not significant. **F** Overexpression of HA-tagged ubiquitin in HEK-293 T cells and analysis of the ubiquitylation of Miwi with or without Ck137956 overexpression by immunoprecipitation using anti-HA antibody. The cells were harvested after a 6-h treatment with 20 μM of MG132. **G** Seminiferous tubule sections were stained with Miwi (red) and DAPI (blue). Pachytene spermatocytes and round spermatids were indicated by red dotted box and solid box, respectively. Scale bar: 20 μm. **H** Venn diagram showing the overlap among down-regulated genes in *Ck137956*^*−/−*^ and *Miwi*^*−/−*^ RS, and target genes of Miwi

To further clarify the function of Ck137956, we analyzed its interacting proteins by immunoprecipitation followed by liquid chromatography-tandem mass spectrometry (IP-LC–MS/MS) (Additional file 1: Fig. S3A). We identified Miwi, a down-regulated protein in *Ck137956*^*−/−*^ spermatids according to the above TMT-based proteomics profiling. Miwi is a cytoplasmic RNA-binding protein whose deletion caused a similar arrest at the round spermatid stage [11]. To confirm the interaction between Ck137956 and Miwi, we overexpressed HA-tagged Ck137956 and Flag-tagged Miwi in HEK-293 T cells, and performed reciprocal co-immunoprecipitation analysis followed by Western blot. The results indicated that Ck137956 interacted with Miwi (Fig. 4A). Furthermore, we also performed reciprocal co-immunoprecipitation assays in testis lysates with or without RNase A/T treatment. We found that Ck137956 interacted with Miwi in testis independent of RNA (Fig. 4B).

To determine which specific domains mediated the interaction between Ck137956 and Miwi, we analyzed protein domain relationships between Ck137956 and Miwi using co-immunoprecipitation assays. Our structural analysis showed that Ck137956 possessed an N-terminal disordered region of 21 amino acids and C-terminal disordered region of 30 amino acids. Miwi contains an N-terminal domain of RG/RA repeats that have been shown to interact with Tudor domain and potentially small RNA processing machinery Dicer [26, 27]. Its central PAZ domain was associated with the 2’-O-methylated 3’-ends of piRNAs, and its MID-PIWI domain at the C-terminal region was reported to anchor its binding to 5’-ends of piRNAs [28]. According to these structural features, we segregated Ck137956 and Miwi into different domains, and overexpressed these truncations followed by co-immunoprecipitation assays. The results showed that Ck137956 interacted with Miwi through its N-terminal domain containing the disordered region and middle part of the C-terminal region (298–447 aa), while Miwi interacted with Ck137956 through domains at both N- and C-terminal regions (Fig. 4C).

Next, we studied the regulatory effects of Ck137956 deletion on Miwi protein. Western blot analysis showed that the protein level of Miwi decreased (Fig. 4D) in 24-day *Ck137956*^*−/−*^ testis, consistent with its down-regulated expression level in *Ck137956*^*−/−*^ round spermatids by proteomic analysis. However, the mRNA level of Miwi remained unchanged after Ck137956 deletion (Fig. 4E). The down-regulation of Miwi protein may be caused by increased degradation by the ubiquitination-proteasome pathway. We expressed HA-tagged ubiquitin in HEK-293 T cells and analyzed the ubiquitination levels of Miwi with or without Ck137956 overexpression after inhibiting proteasome activity by MG132 treatment. We observed that Miwi had a lower ubiquitination level after Ck137956 overexpression (Fig. 4F), suggesting that Ck137956 increased the stability of Miwi by down-regulating its ubiquitination level.

Similar to Ck137956, Miwi is also expressed in the cytoplasm of pachytene spermatocytes and the round spermatids [11]. In round spermatids, Miwi is localized in the chromatoid body (CB), a single lobulated perinuclear granule involved in RNA processing [29]. To detect the CB structure in *Ck137956*^*−/−*^ mice, we performed the TEM and observed that the CB structure was not affected after *Ck137956* deletion (Additional file 1: Fig. S3B). Consistently, MVH, a component of CB proteins, was normally enriched at the CB in *Ck137956*^*−/−*^ round spermatids (Additional file 1: Fig. S3C). However, deletion of Ck137956 disrupted the localization of Miwi to dotted CB in round spermatids, while the cytosolic localization in pachytene spermatocytes was not altered (Fig. 4G). To evaluate the CB localization of Ck137956, we isolated CB fractions with anti-MVH antibody by immunoprecipitation using the protocol reported previously [30]. Western blot results showed that Ck137956 (Additional file 1: Fig. S3D) existed the CB fraction. Furthermore, we compared the previously reported down-regulated genes in *Miwi*^*−/−*^ round spermatids [31] with the down-regulated genes in *Ck137956*^*−/−*^ round spermatids. Venn diagram results showed that 436 (54%) down-regulated genes in *Miwi*^*−/−*^ round spermatids were also down-regulated in *Ck137956*^*−/−*^ round spermatids (Fig. 4H). Among the 71 down-regulated genes bound with Miwi reported by Vourekas, A, et al. using HITS-CLIP [16], 58 (81%) were down-regulated in *Ck137956*^*−/−*^ round spermatids. Thus, Ck137956 regulated the localization and stability of Miwi in round spermatids, and may regulate the stability of Miwi-bound genes.

### Ck137956 regulates the mRNA-stabilizing activity of Miwi

**Fig. 5:**
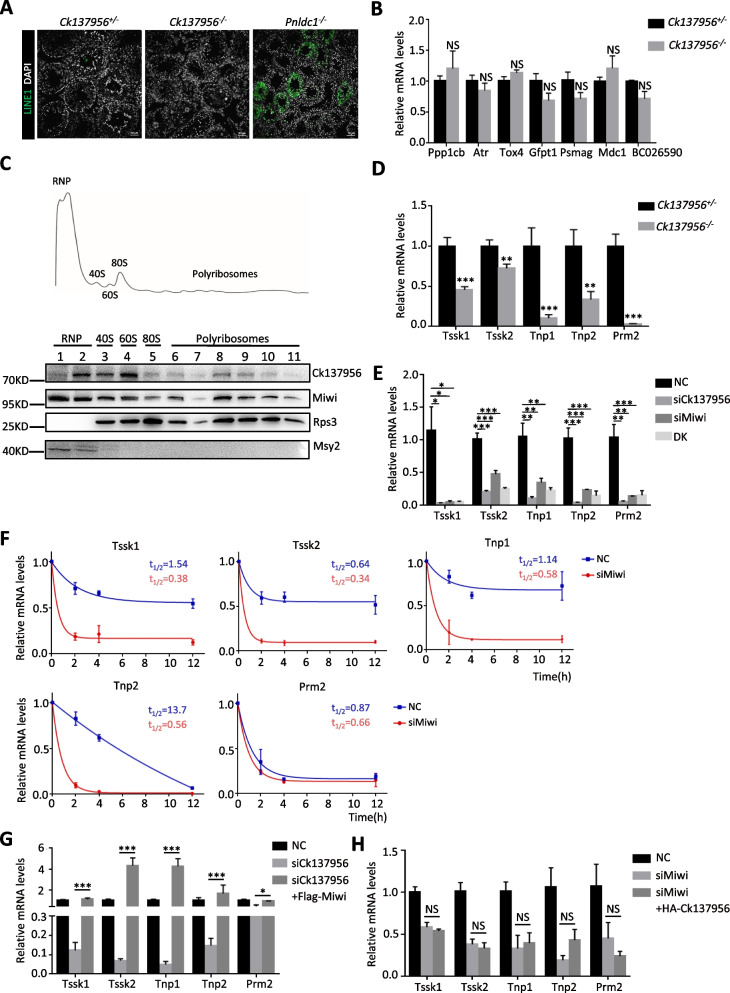
Ck137956 alters the mRNA-stabilizing ability of Miwi. **A** Immunofluorescence analysis of LINE1 (green) in *Ck137956*^+/-^, *Ck137956*^−/−^ and *Pnldc1*^−/−^ testes. DAPI (white) stained the nuclei. Scale bars: 50 μm. **B** qRT-PCR analysis of the mRNA expression levels of *Ppp1cb, Atr, Tox4, Gfpt1, Psmag, Mdc1* and *BC026590* in *Ck137956*^−/−^ and control round spermatids. *n* = 3. **C** Distribution of Ck137956 and Miwi proteins in different fractions collected from sucrose gradient sedimentation of testis lysate. Rps3 and Msy2 were used as mono/polysomes and mRNPs controls, respectively. **D** qRT-PCR analysis of *Tssk1*, *Tssk2*, *Tnp1*, *Tnp2*, *Prm2* mRNA expression levels in mRNPs of *Ck137956*^+/-^ and *Ck137956*^*−/−*^ testes. *n* = 9. **E** qRT-PCR analysis of mRNAs expression levels in GC-2spd(ts) cells treated with siCk137956 or/and siMiwi with scrambled sequence as a control. DK, doubleknock down group. The statistical significance between the groups was plotted. *n* = 3. **F** Quantitation of mRNA stability in siMiwi-treated and control GC-2spd(ts) cells after Actinomycin D treatment (10 μg/ml). The upper right corner shows the half-life time (t_1/2_) of gene expression in siMiwi-treated and control cells. *n* = 3. **G** qRT-PCR analysis of mRNA expression in siCk137956-treated cells with or without Miwi overexpression. *n* = 3. **H** qRT-PCR analysis of mRNA expression levels in siMiwi-treated cells with or without Ck137956 overexpression. *n* = 3. All data were represented as mean ± SEM. NS, not significant, *p < 0.05, **p < 0.01, ***p < 0.001

As the Piwi-interacting RNAs (piRNAs) binding protein, Miwi/piRNAs can silence retrotransposons such as LINE1 [31] and degrade mRNA [32]. To evaluate the changes of piRNA in *Ck137956*^*−/−*^ testis, we selected a few piRNAs previously reported to be bound by Miwi [16] for qRT-PCR analysis. The results showed that the expression levels of these piRNAs were unaffected after deletion of Ck137956 (Additional file 1: Fig. S4A). In addition, piRNAs are abundant in chromatoid bodies (CBs) [30]. And CB fragmentation is a common defect associated with piRNA deficiency in piRNA-deficient mutant mice [33, 34]. However, the intact CB structure was observed in *Ck137956*^*−/−*^ round spermatids (Additional file 1: Fig. S3B), which suggesting that deletion of Ck137956 might not affect the piRNA levels. Next, we performed immunofluorescence analysis of LINE1 in *Ck137956*^*−/−*^ testis (Fig. 5A) with *Pnldc1*^−/−^ testis as a positive control. The results showed that LINE1 was derepressed as expected in *Pnldc1*^−/−^ testis [35], but remained repressed in *Ck137956*^*−/−*^ testis, indicating the intact function of Miwi/piRNA in retrotransposon repression. As to the mRNA degradation function of Miwi/piRNA, we selected seven target genes reported to be upregulated in *Miwi*^*−/−*^ round spermatids, including Ppp1cb, Atr, Tox4, Gfpt1, Psmag, Mdc1 and BC026590 [14], for qRT-PCR analysis. We found that all these seven genes were unchanged in *Ck137956*^*−/−*^ round spermatids (Fig. 5B), which demonstrated that the mRNA degradation function of Miwi/piRNA was unaffected after *Ck137956* deletion.

In light of numerous mRNAs down-regulated without change of the pre-mRNAs in *Ck137956*^*−/−*^ testis, these mRNAs may have decreased stability. Studies have shown that Miwi may form cytoplasmic mRNPs, which are devoid of piRNAs, and stabilize spermiogenic mRNAs in a piRNA-independent manner [16]. After *Ck137956* deletion, Miwi is not localized in chromatoid body, a specialized assembly of cytoplasmic mRNP foci present in germ cells, which often are implicated in storage and localization of mRNAs [36, 37]. To analyze the roles of Ck137956 in mRNPs, we resolved adult mouse testis lysates by ultracentrifugation in a sucrose density gradient (Fig. 5C). The distinct distribution of repressed mRNPs protein Msy2 in mRNPs, and ribosomal protein Rps3 in ribosome fractions demonstrated the successful separation of various macromolecular complexes. Western blot results showed that Miwi was present in both the repressive mRNPs and ribosome fractions in line with the previous report [16]. The presence of Ck137956 in mRNPs indicated that Ck137956 and Miwi might function together in stabilizing spermiogenic mRNAs in mRNPs. Furthermore, we quantified the expression levels of spermiogenic genes bound by Miwi based on HITS-CLIP analysis [16] including *Tssk1, Tssk2, Tnp1, Tnp2 and Prm2*. The results showed that their levels in mRNPs factions were significantly decreased after the deletion of Ck137956 (Fig. 5D). Ck137956 may regulate the stability of Miwi-interacting spermiogenic mRNAs in mRNPs.

To further characterize the mechanisms of Ck137956/Miwi in mRNA stability regulation, a male mouse spermatocyte-derived GC-2spd(ts) cell line [38], an in vitro system usually used in functional studies of Miwi [15, 39], was used. We found that Ck137956, Miwi, and known target genes bound by Miwi were expressed in GC-2spd(ts) (Additional file 1: Fig. S4B). To evaluate effects of Miwi and/or Ck137956 on the stability of those target genes, we knocked down endogenous Miwi and/or Ck137956 using siRNA in GC-2spd(ts) cells. With validated knockdown effects of siMiwi and siCk137956 (Additional file 1: Fig. S4C-D), we examined the expression levels of endogenous *Tssk1, Tssk2, Tnp1, Tnp2, Prm2* in GC-2spd(ts). The results showed that knockdown of either gene and both genes resulted in similar decrease in expression of target genes (Fig. 5E) compared to the control. Next, we analyzed the half-life of mRNA as a measure for mRNA stability by Actinomycin D treatment in siMiwi-treated and control GC-2spd(ts) cells. We found that Miwi knockdown led to shorter half-lives and lower stabilities of the Miwi target genes compared with the controls (Fig. 5F). Moreover, we analyzed the stabilities of target genes by normalization against 0 h after 4 h-actinomycin D treatment. The results showed that knockdown of either gene and both genes led to decreased stability of target genes to a similar level (Additional file 1: Fig. S4E) and resulted in similar effect on mRNA stability, indicating that Ck137956 and Miwi may participate in the same pathway. Furthermore, Miwi overexpression in siCk137956-treated cells can rescue the decrease of mRNA stability of Miwi target genes (Fig. 5G), while Ck137956 overexpression in siMiwi-treated cells had no such effect (Fig. 5H). Therefore, Ck137956 is upstream to Miwi, and can regulate the mRNA stabilizing function of Miwi. Ck137956/Miwi play important functions in post-transcriptional regulation of spermiogenesis-related genes.

## Discussion

In this study, we identified a novel germ cell-specific cytoplasmic post-transcriptional regulator, Ck137956 in sperm formation. Our data demonstrated that Ck137956 was a testis-specific gene, and it was essential for male fertility and spermiogenesis of the round spermatids beyond step 6. Thus, we propose “Tssa” (testis-specific and spermiogenic arrest gene) as a novel name for Ck137956. Tssa interacted with Miwi, affected the localization and stability of Miwi in round spermatids and regulated the level of target mRNAs of Miwi in repressed mRNPs during spermatogenesis. Its deletion leads to the destabilization of target genes of Miwi at mRNA levels.

We found that deletion of Tssa caused round spermatid arrest. During spermiogenesis, the transformation of round spermatids into elongated spermatids requires complex transcriptional and post-transcriptional regulations [40]. Studies have shown that the nuclear protein Sox30 is a transcriptional factor, and its deletion also caused spermatogenic arrest at the round spermatid stage [41]. The deficiency of GRTH, an RNA-binding protein involved in mRNA shuttling and translational stimulation, causes an arrest in late (step 8) round spermatids [42, 43]. Tssa is conserved in mammals. We find that Tssa is also essential for round spermatid development. Numerous mRNAs associated with spermiogenesis are down-regulated after deletion of Tssa. Our data demonstrate that Tssa is involved in stabilizing these down-regulated mRNAs by interacting with Miwi.

We identified Tssa as an important Miwi-interacting protein, which regulated the mRNA stabilizing function of Miwi. Miwi is a germline-specific Argonaute family member and its deletion also leads to the arrest of round spermatids. Miwi, as an RNA-binding protein, regulates the activity of transposon through its binding piRNAs, and is known to bind mRNAs. Various germline Tudor proteins (Tdrd1, Tdrkh, Tdrd6, Tdrd7, and Stk31) are in complex with Miwi in germ cells, indicating the biochemical and functional link between Miwi and the germline Tudor proteins [26, 27]. Mutations of Miwi are known to cause male infertility in humans [44]. Although the functions of Miwi are important, its regulators are still not well known. We found that Tssa deletion affected the chromatoid body localization of Miwi in round spermatids. Tssa deletion up-regulated the ubiquitination level and down-regulated the expression level of Miwi. The elucidation of the regulation of Miwi is expected to help better understand the molecular mechanisms of spermiogenesis and male infertility.

We find that Tssa/Miwi regulate the stability of spermiogenic mRNAs, including Tssk1, Tssk2, Tnp1, Tnp2 and Prm2. These genes are all subjected to uncoupling of transcription and translation during spermiogenesis. In spermatogenesis, multiple specific spermiogenic mRNAs are transcribed in advance and stored in mRNPs to be actively translated in the specific period. Translation can be repressed by specific sequences to block the assembly of the translation complex [45]. FXR1 can convert stored mRNAs into a translationally activated state by liquid–liquid phase separation (LLPS) [46]. Translation of mRNAs can be activated by MIWI/piRNA at a specific window during spermiogenesis [15]. However, how these repressed mRNAs are stabilized is still not well unknown. Miwi can bind and stabilize certain spermiogenic mRNAs in mouse testes in a piRNA-independent way [16]. Our findings reveal that the mRNA stability function of Miwi is regulated by Tssa. Both Tssa and Miwi are localized in repressed mRNPs and collaborate together to stabilize translationally repressed mRNAs.

## Conclusions

In conclusion, we identified a new Miwi regulator, Tssa. Similar to Miwi, the deletion of Tssa leads to arrest at round spermatids. Tssa regulates the localization and stability of Miwi and interacts with Miwi to regulate the stability of spermiogenesis-related genes. The diverse functions of Miwi may be regulated by different upstream factors, which deserve further studies.

### Methods

All siRNA oligonucleotide sequences, primers for qRT-PCR and antibodies are given in Additional file 2: Table S1, S2, S3 and S4.

### Generation of Ck137956 knockout mouse models

All animal experiments were approved by the Institutional Animal Care and Use Committee of Nanjing Medical University and in accordance with the guidelines and regulations of the Committee. The Ck137956 knockout mice were generated by the CRISPR/Cas9 technology. The single guide RNAs (sgRNAs) were designed to target exon 3 of Ck137956. The Cas9 mRNA and sgRNAs were transcribed in vitro and microinjected into the cytoplasm of C57BL/6 J mouse zygotes. The injected embryos were implanted into pseudo-pregnant mice according to standard procedures to obtain the founder mice.

### Cell culture and transfections

The HEK-293 T and GC-2spd (ts) cells were used in this study. Cells were cultured in DMEM supplemented with 10% FBS, penicillin (100 U/ml) and streptomycin (100 g/ml) in 37 °C and 5% CO_2_. Transfections were performed with ExFect Transfection Reagent (Vazyme) in HEK-293 T cells or Lipofectamine 3000 (Invitrogen) in GC-2spd (ts) cells according to the manufacturer's protocol.

### Plasmids and siRNA oligonucleotides

Ck137956, Miwi and Ubiquitin were amplified by PCR using adult mouse testis cDNA, tagged with HA, Flag or V5, and inserted into pCDNA3.1 linearized vector via homologous recombination. All DNA plasmids were endotoxin free, and were used to transfect HEK-293 T or GC-2spd (ts) cells. siRNA oligonucleotides against Miwi were synthesized by GenePharma (China), and siRNA oligonucleotides against Ck137956 by Dharmacon (America). Their sequences are listed in the Additional file 2: Table S1.

### Antibody

The rabbit Ck137956 antibody was generated in Abclonal (Wuhan, China). Briefly, polyclonal antibody was produced by immunizing rabbits with a synthetic peptide KLPESSPPKROLPVFAKIC corresponding to the 301-319aa of mouse Ck137956 protein, and was then purified by affinity chromatography.

### Phylogenetic analysis

Orthologs of *Ck137956* were retrieved from the homologene database. The phylogenetic tree of the orthologs of Ck137956 from various species was constructed using the neighbor-joining method with a bootstrap of 1000 by MEGA11 software [47].

### Isolation of spermatogenic cells

Using STA-PUT method described previously [41, 48], pachytene spermatocytes, round spermatids and elongated spermatids were isolated from adult mouse testes, and spermatogonia were isolated from postnatal day 8 mouse testes. Briefly, the testes were digested into single-cell suspensions in a two-step process: i) collagenase IV (1 mg/ml) at 37 °C for 10 min; ii) 0.25% trypsin with DNase I (2 mg/ml) at 37 °C for 10 min. The single-cell suspension was loaded into a cell separation device (ProScience Inc. Canada) and separated by a 2–4% bovine serum albumin (BSA) gradient formed by 2% BSA and 4% BSA in DMEM. Spermatogenic cells were harvested after 2 h sedimentation for follow-up tests. For isolation of Sertoli cells [49], single-cell suspensions of adult testes using the above two-step process were cultured in cell culture dishes for 24 h. Sertoli cells adhering to the bottom of the dish were collected and washed by DPBS (Gibco) for later analysis.

### Histological and electron microscopic analysis

The testes and epididymis were fixed in modified Davidson fluid (MDF) for 48 h, dehydrated with gradient ethanol (70%, 80%, 90% and 100%), cleared in xylene, and embedded in paraffin. For histological examination, sections were then cut at 5-μm thick and stained routinely with Hematoxylin and Eosin.

For electron microscopy, samples were fixed in 2.5% glutaraldehyde at 4 °C overnight, dehydrated through increasing concentration of ethanol (30%, 50%, 70%, 90% and 100%), embedded in Epon812 (Sigma) and polymerized. After ultrathin sectioning, sections were stained with uranyl acetate and lead citrate, and then analyzed by transmission electron microscope (TEM) (JEOL, JEM-1010).

### Immunofluorescent staining, TUNEL assay and chromosome spread

For immunofluorescent staining, paraffin sections were boiled in heat-induced antigen retrieval buffer (1.8 mM citric acid, 8.2 mM sodium citrate, pH 6.0), washed with 0.1% PBS-Triton-X-100 (PBST), blocked with 5% BSA for 2 h at room temperature, and then incubated with primary antibodies at 4 °C overnight. TUNEL assay was performed using TUNEL BrightGreen Apoptosis Detection Kit (Vazyme) according to the manufacturer's instruction. Chromosome spread were performed as previously described [50, 51]. Testicular cell suspension were prepared in hypotonic buffer [30 mM Tris–HCl pH 8.5, 50 mM sucrose, 17 mM sodium citrate, 5 mM EDTA, 50 mM DTT, 10 mM PMSF] for 30 min at room temperature, and cell suspension were then dropped onto the paraformaldehyde solution-coated slides, which were air-dried and further incubated with primary antibodies for immunofluorescent staining.

### Immunoblotting

Protein extracts were prepared using iced RIPA lysis buffer (Beyotime) containing 1 × protease inhibitor cocktail (Biomake), separated via SDS-PAGE, and transferred onto PVDF membranes. The membranes were blocked with 5% non-fat milk for 2 h at room temperature, and incubated with primary antibodies overnight at 4 °C. After washed by 0.1% TBS-Tween-20 (TBST) for three times, the membranes were incubated with secondary antibodies at room temperature for 2 h. Specific protein bands were then visualized with the High-sig ECL western blotting substrate (Tanon).

### Quantitative RT-PCR (qRT-PCR) and RNA-seq

Total RNA in cells or tissues were extracted with RNAiso Plus (Takara), reverse transcribed using PrimeScriptRT Master Mix (Takara), and subsequently used for quantitative RT-PCR by AceQ qPCR SYBR Green Master Mix (Vanzyme) on a QuantStudio 7 (Applied Biosystems) system according to the manufacturer's instruction. qRT-PCR data were analyzed by the comparative ΔΔCt (cycle threshold) method, which allowed us to calculate the change in relative gene expression. For RNA-seq analysis, strand-specific libraries were prepared by the TruSeq Stranded Total RNA Sample Preparation kit (Illumina) according to the manufacturer's instructions and then sequenced by the Illumina Nova6000 system. Clean data were obtained by trimming the adaptor sequences and removing low quality sequences. Clean reads were then mapped to the mouse genome (mm10). The aligned reads of genes were counted to assess gene expression levels after normalization by DESeq2. A gene with adjusted *p* < 0.01 and Log_2_FoldChange > 2 or Log_2_FoldChange < -2 was considered significant.

### Protein sample preparation and TMT labelling

Round spermatids were lysed by protein extraction buffer [8 M Urea, 75 mM NaCl, 50 mM Tris, pH 8.2, 1% (vol/vol) EDTA-free protease inhibitor, 1 mM NaF, 1 mM β-glycerophosphate, 1 mM sodium orthovanadate, 10 mM sodium pyrophosphate] at 4 °C, reduced, trypsin digested, and desalted using Waters Co. (Milford, MA) Sep-Pak column. For TMT labelling, purified peptides were reconstituted in 200 mM triethylammonium bicarbonate (TEAB) and labelled with TMT-6plex (Thermo) according to the manufacturer’s instructions. After incubation for 1 h at room temperature, the reaction was quenched for 15 min by 5% hydroxylamine [52]. All six samples were combined and purified using an OASIS HLB 1-cc Vac cartridge (Waters) and then lyophilized for subsequent analysis.

### High-pH reverse phase fractionation

The TMT-labelled peptides were fractionated by high-pH reverse phase column as previously described [53] using a Xbridge™ BEH130 C18 column (2.1 × 150 mm, 3.5 μm, Waters) with the UltiMate® 3000 HPLC systems. Buffer A (10 mM ammonium acetate, pH 10) and buffer B (90% ACN/10 mM ammonium acetate, pH 10) were employed under a 60 min gradient (0%–7% buffer B for 3 min, 7%–42% B for 40 min, 42%–70% B for 12 min, followed by 5 min at 70% B). Fractions were collected and dried with a SpeedVac concentrator.

### Mass spectrometry analysis

Thirty fractions of peptides were reconstituted in 0.1% FA and submitted to LC–MS/MS analysis using an Easy-Nlc 1000 system with trap column (75 μm × 2 cm, Acclaim® PepMap100, Thermo) and analytical column (75 μm × 25 cm, Acclaim® PepMap RSLC, Thermo) in flow rate of 300 nl/min. Mass spectrometry (MS) acquisition was performed in a data-dependent mode using a 205 min gradient (3% to 8% buffer B for 3 min, 8% to 29% buffer B for 176 min, 29% to 41% buffer B for 15 min, 41% to 90% buffer B for 1 min, 90% buffer B for 10 min) on a LTQ Orbitrap Velos [54]. An MS survey scan was obtained, and a low-energy MS/MS scan of every precursor in the linear ion trap (collision induced dissociation, CID) followed by a higher energy MS/MS scan in the octopole collision cell (higher energy collision dissociation, HCD) was acquired from the survey scan for the eight most intense ions.

Raw files were searched against mouse protein sequences obtained from the Universal Protein Resource database (UniProt, release 2022_01) using MaxQuant software (1.6.5.0). The FDR cut-off was set to 0.01 for proteins and peptides. Full cleavage by trypsin, and a maximum of two missed cleavage sites was used. Carbamidomethyl (C) on cysteine-fixed modifications, with TMT reagent adducts on lysine and peptide amino termini, was considered fixed modifications. Variable modifications included oxidation (M) and acetylation (protein N-term). The corrected TMT reporter intensities were used for TMT-based protein quantification. A protein with *p* value < 0.001 (Student’s t-test) was considered significantly differentially expressed.

### GO enrichment analysis

GO enrichment analysis were performed by the “ClusterProfiler” package [55] and visualized by the “ggplot2” package in the R software. A term with adjusted *p* < 0.05 was considered significant.

### Sucrose density gradient analysis

Sucrose density gradient analysis was performed as previously described [16] with minor modifications. In brief, testicular tissues were homogenized in MCB Buffer [100 mM KCl, 2 mM MgCl2, 10% glycerol, 50 mM HEPES, 0.1% Triton X-100, 1 mM DTT, 20 unit/ml RNase Inhibitor, 100 μg/ml cycloheximide (CHX)], and centrifuged at 12 000 rpm for 15 min to remove nuclear pellets. A 20%-50% sucrose density gradient was prepared in advance. The extracts were then laid over the gradient and centrifuged at 38 000 rpm for 2.5 h at 4 °C. Each fraction collected was subjected to analysis of indicated proteins and RNAs by western blotting and qRT-PCR.

### Co-immunoprecipitation

Testicular tissues were lysed in cold IP lysis buffer (Thermo) with 1 × protease inhibitor cocktail (Biomake), incubated for 30 min at 4 °C, and centrifuged at 16 000 g for 30 min at 4 °C. The supernatant was pre-cleared using Protein A/G beads (Millipore) at 4 °C for 2 h, and then incubated with antibody-coupled magnetic beads overnight at 4 °C. Beads were washed for four times with wash buffer [20 mM Tris, pH 7.4, 150 mM NaCl, 0.5% Triton X-100, 1 mM EDTA], and bound proteins were eluted with sample buffer containing 1% SDS for 10 min at 95 °C, and detected by immunoblotting.pCDNA3.1-HA-Ck137956 and pCDNA3.1-Flag-Miwi plasmids were co-transfected into HEK-293 T cells. After 48 h transfection, cells were lysed in IP lysis buffer (Thermo) and 1 × protease inhibitor cocktail (Biomake) for 20 min at 4 °C and centrifuged at 12 000 g for 20 min at 4 °C. The cell lysates were incubated with anti-HA Magnetic Beads (Thermo) or anti-DYKDDDDK Resin (GenScript) for 12 h at 4 °C, and washed with wash buffer. Beads or resin were finally eluted with sample buffer containing 1% SDS at 95 °C, and analyzed using immunoblotting.

### Immunoprecipitation and mass spectrometry

Testicular tissues were lysed in cold Pierce™ IP lysis buffer (Thermo) in the presence of 1 × protease inhibitor cocktail (Biomake), revolved for 1 h at 4 °C and centrifuged at 40 000 g for 1 h. After being pre-cleared with protein A/G magnetic beads (Millipore) at 4 °C for 1 h, the lysates were incubated with anti-Ck137956 antibody-coupled magnetic beads overnight at 4 °C. Beads were finally eluted with sample buffer containing 1% SDS at 95 °C for 10 min. The eluted proteins were subjected to silver stain and mass spectrometry (MS). For MS analysis, silver stained gel lanes were cut into small pieces, digested with trypsin overnight with 0.4% trifluoroacetic acid to stop digestion. The peptides were desalted, re-suspended in 0.1% formic acid (v/v) as described previously [56], and analyzed using an Orbitrap Fusion Lumos mass spectrometer (Thermo).

### Actinomycin D assay

The Actinomycin D assay was performed as previously described [57]. Briefly, Miwi siRNA were transfected into GC-2spd (ts) cells. Forty-eight hours after transfection, cells were subjected to Actinomycin D (10 μg/ml, MCE) for 0 h,2 h,4 h,12 h. Total RNA were extracted from each time point. The mRNA expression levels were measured through qRT-PCR assay.

### piRNA expression analysis

Total RNAs were extracted with RNAiso Plus (Takara) and reverse transcribed into cDNA using miRNA All-In-One cDNA Synthesis Kit (abm, Canada). Universal 3' miRNA Reverse Primer (abm) and specific forward primers were used for qRT-PCR analysis in quantitation of piRNA expression levels.

### Statistical analysis

All data were presented as the mean ± SEM values. Two-tailed Student’s t test or one way ANOVA with Dunnett’s t test was used to determine the statistical significance. *p*-values < 0.05 were considered significant.

## Supplementary Information

**Additional file 1: Fig. S1.** Phenotype analysis of *Ck137956*^-/-^ mice. **Fig. S2.** Gene ontology enrichment analysis of down-regulated genes in* Ck137956*^-/-^ pachytene spermatocytes. **Fig. S3.** Analysis of Ck137956-interacting protein Miwi. **Fig. S4. **Verification of siRNA knock down efficiency.

**Additional file 2: Table S1.** siRNA oligonucleotides. **Table S2.** Gene-specific primers used in qRT-PCR. **Table S3.** piRNA specific primers used in qRT-PCR. ** Table S4.** Antibodies used in this study.

**Additional file 3.** RNA-seq profiling of *Ck137956*^*+/-*^ and *Ck137956*^*-/-*^ pachytene spermatocytes.

**Additional file 4.** RNA-seq profiling of *Ck137956*^*+/-*^ and *Ck137956*^*-/-*^ round spermatids.

**Additional file 5.** Proteomic profiling of *Ck137956*^*+/-*^ and *Ck137956*^*-/-*^ round spermatids.

**Additional file 6.** Includes individual values depicted in Figs. 1, 2, 3 and 5.

**Additional file 7.** Includes individual values depicted in Additional file 1: Figure S1 and S4.

**Additional file 8.** Uncropped gels/blots.

## Data Availability

All data generated during this study are included in this published article, its supplementary information files and publicly available repositories. The mass spectrometry proteomics data are available on the ProteomeXchange Consortium (http://proteomecentral.proteomexchange.org) by the iProX [58] partner repository with the identifier PXD036804. The RNA-seq data are deposited in the NCBI database under the accession number PRJNA862707. Publicly available datasets analysed during the current study are available in the GEO database under accession codes GSE32180 https://www.ncbi.nlm.nih.gov/geo/query/acc.cgi?acc=GSE32180 [31]. The individual data values for Figs. 1, 2, 3 and 5 are provided in Additional file 6. The individual data values for Additional file 1: Figure S1 and S4 are provided in Additional file 7. The uncropped gels/blots are provided in Additional file 8.

## Abbreviations

mRNPs: Messenger ribonucleoproteins
Prm1: Protamine 1
Prm2: Protamine 2
PNA: Peanut agglutinin
piRNAs: Piwi-interacting RNAs
TEM: Transmission electron microscopy
GO: Gene Ontology
CC: Cellular component
BP: Biological processes
TMT: Tandem mass tags
LC–MS/MS: Labelling-based tandem mass spectrometry
CB: Chromatoid body
LLPS: Liquid–liquid phase separation
