# Supplementary figures and images for: Regulation of Miwi-mediated mRNA stabilization by Ck137956/Tssa is essential for male fertility
Source: BMC Biol. 2023 Apr 17;21:89. doi: 10.1186/s12915-023-01589-z (PMC10111675; doi:10.1186/s12915-023-01589-z)

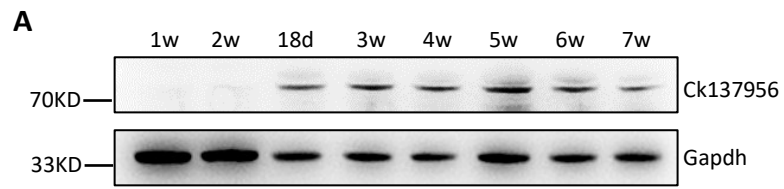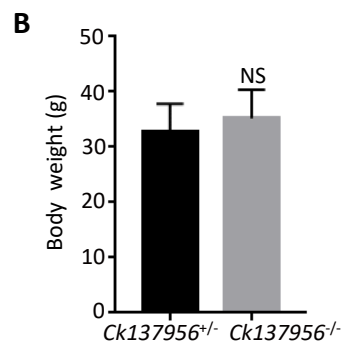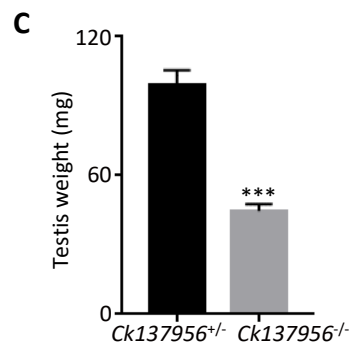

Supplement: Supplementary file 1 — Additional file 1: Fig. S1. Phenotype analysis of Ck137956-/- mice. Fig. S2. Gene ontology enrichment analysis of down-regulated genes in Ck137956-/- pachytene spermatocytes. Fig. S3. Analysis of Ck137956-interacting protein Miwi. Fig. S4. Verification of siRNA knock down efficiency. [file 12915_2023_1589_MOESM1_ESM.zip › Additionl file 1_Figure_S1.pdf]

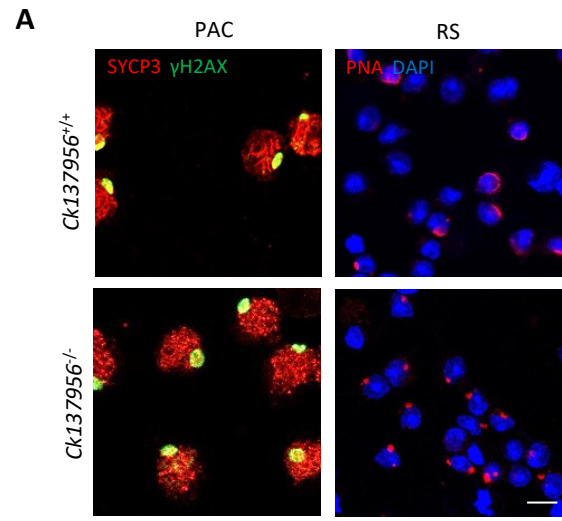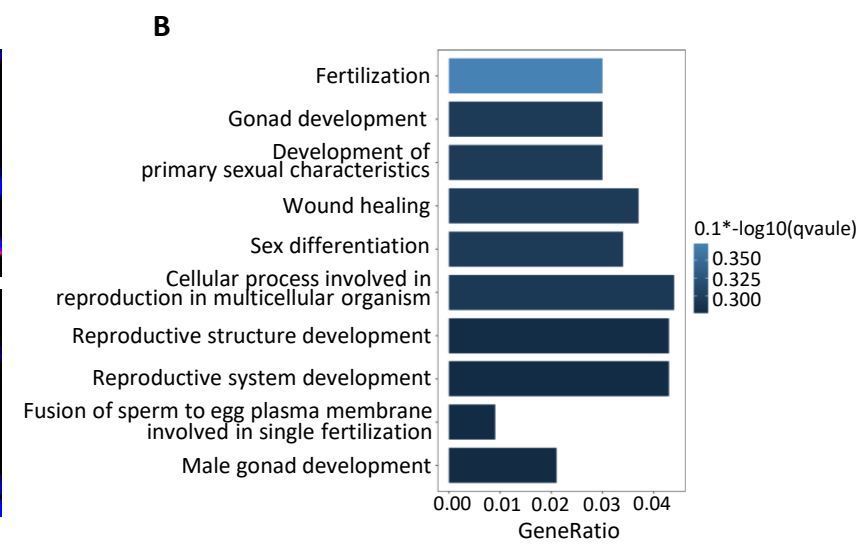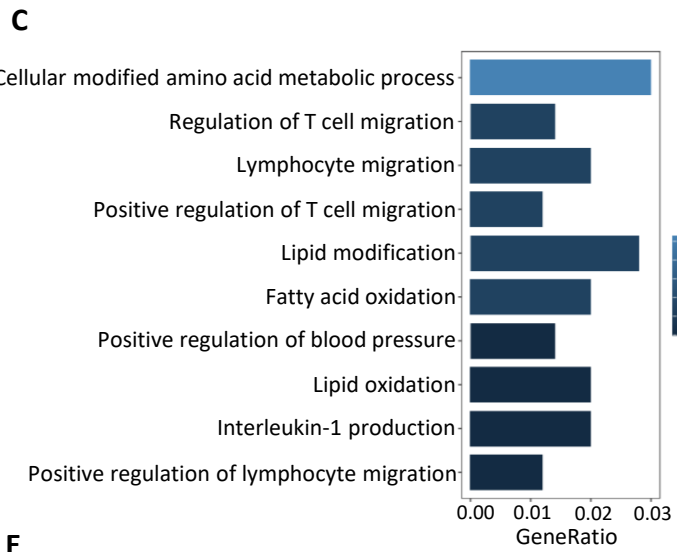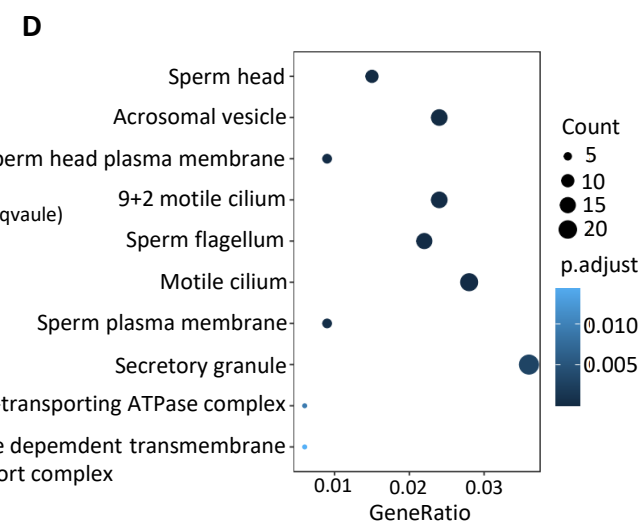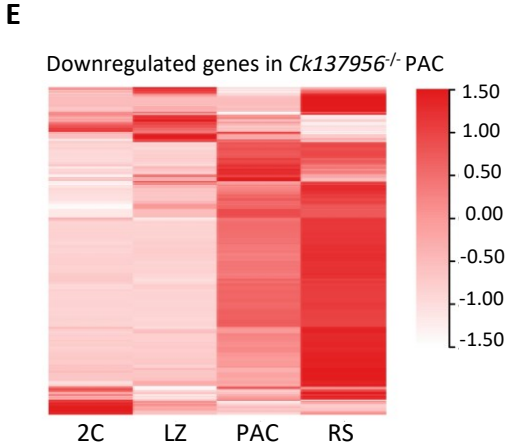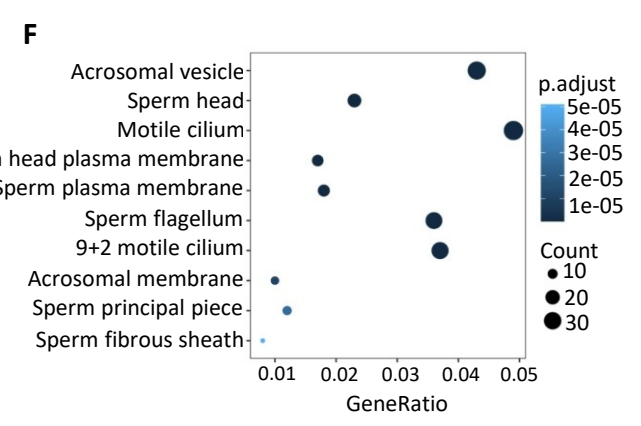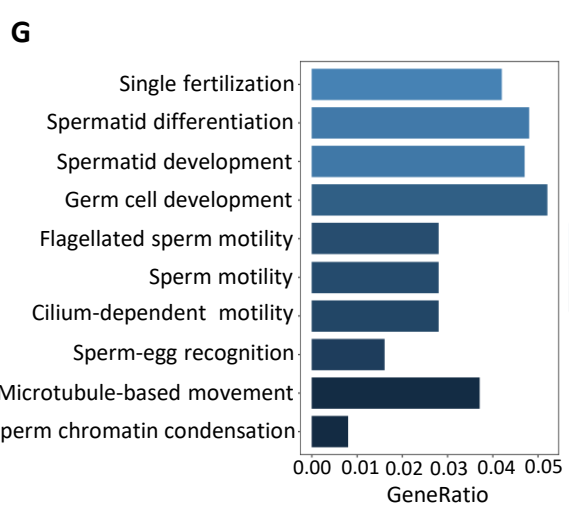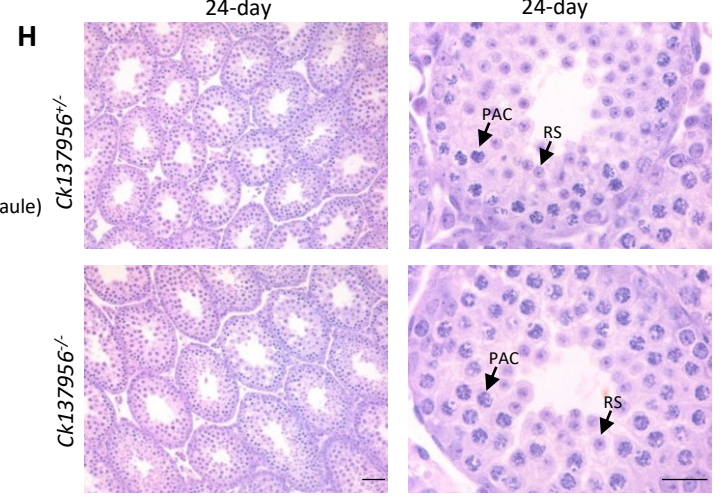

Supplement: Supplementary file 1 — Additional file 1: Fig. S1. Phenotype analysis of Ck137956-/- mice. Fig. S2. Gene ontology enrichment analysis of down-regulated genes in Ck137956-/- pachytene spermatocytes. Fig. S3. Analysis of Ck137956-interacting protein Miwi. Fig. S4. Verification of siRNA knock down efficiency. [file 12915_2023_1589_MOESM1_ESM.zip › Additionl file 1_Figure_S2.pdf]

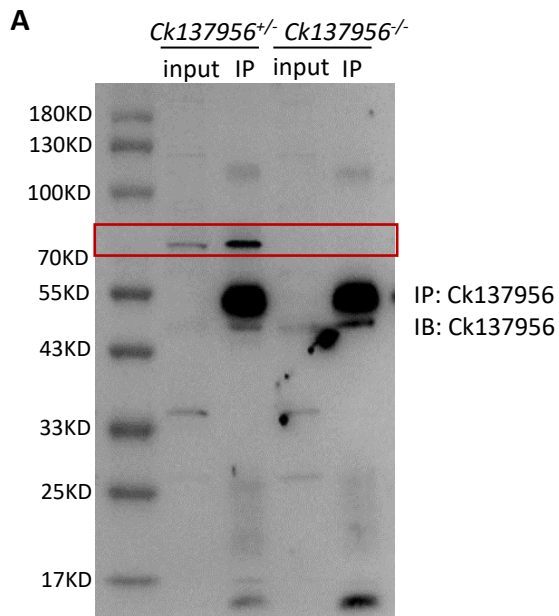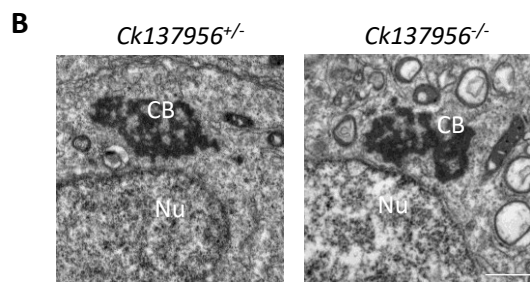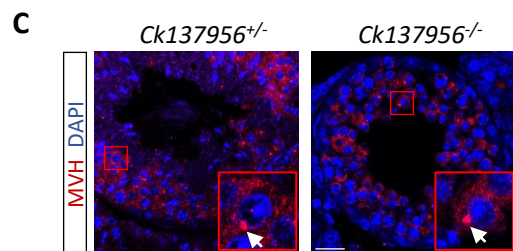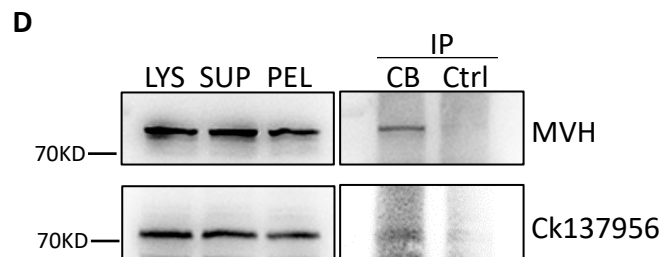

Supplement: Supplementary file 1 — Additional file 1: Fig. S1. Phenotype analysis of Ck137956-/- mice. Fig. S2. Gene ontology enrichment analysis of down-regulated genes in Ck137956-/- pachytene spermatocytes. Fig. S3. Analysis of Ck137956-interacting protein Miwi. Fig. S4. Verification of siRNA knock down efficiency. [file 12915_2023_1589_MOESM1_ESM.zip › Additionl file 1_Figure_S3.pdf]

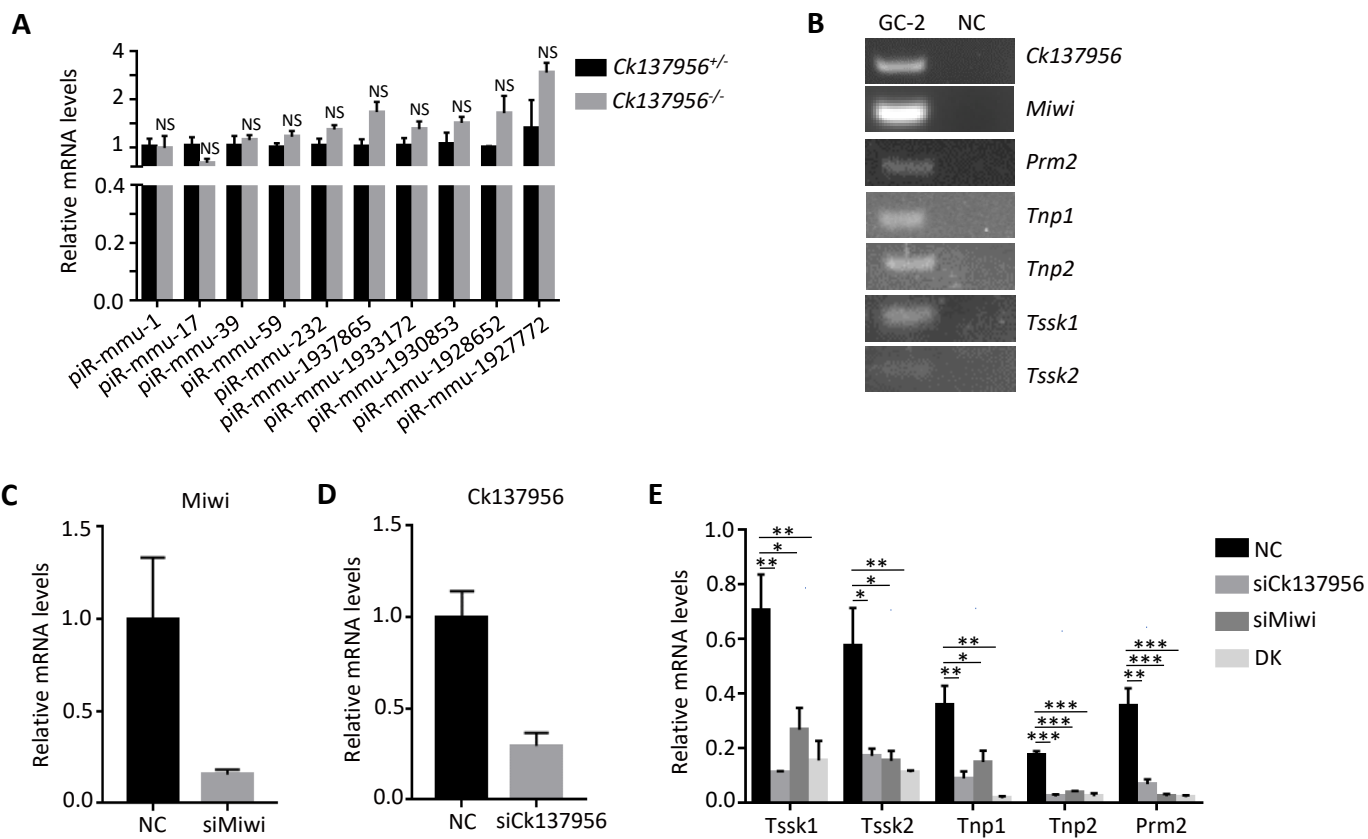

Supplement: Supplementary file 1 — Additional file 1: Fig. S1. Phenotype analysis of Ck137956-/- mice. Fig. S2. Gene ontology enrichment analysis of down-regulated genes in Ck137956-/- pachytene spermatocytes. Fig. S3. Analysis of Ck137956-interacting protein Miwi. Fig. S4. Verification of siRNA knock down efficiency. [file 12915_2023_1589_MOESM1_ESM.zip › Additionl file 1_Figure_S4.pdf]
