# Supplementary material for: Regulation of Miwi-mediated mRNA stabilization by Ck137956/Tssa is essential for male fertility
Source: BMC Biol. 2023 Apr 17;21:89. doi: 10.1186/s12915-023-01589-z (PMC10111675; doi:10.1186/s12915-023-01589-z)
Supplement: Supplementary file 2 — Additional file 2: Table S1. siRNA oligonucleotides. Table S2. Gene-specific primers used in qRT-PCR. Table S3. piRNA specific primers used in qRT-PCR. Table S4. Antibodies used in this study. [file 12915_2023_1589_MOESM2_ESM.pdf]

Table S1. siRNA oligonucleotides

	Sequence(5'to3')
Miwi siRNA	5'-UACUUCUGUGACCUUGUGC-3'
Ck137956 siRNA (SMARTpool)	5'-CGUAAUGGCUGGUGACGAG-3'
	5'-GGGAAUGGCAUACGCUUUU-3'
	5'-CCUACGACAGUGCCCGAAA-3'
	5'-CAGCUGAGGCUUCGAACCA-3'

Table S2. Gene-specific primers used in qRT-PCR

Gene	Forward primer (5'-3')	Reverse primer (5'-3')
Ck137956	GAACCGGAAGGACACCTGTG	GCACTGTCGTAGGGGAGTTAAA
Miwi	CAGCAACCTGGGTACATCCC	CCAAGGTCATGGAAGTCTCGG
Tssk1	CTCAAGCGACGAGGCTACATC	ACCGCCACGTTGAACTTTAGG
Tssk2	GCGGTCCTAAGGAAGAAGGG	TGACTGCCACATTGAACTTGAG
Prm2	ACAAGACCATGAACGCGAGG	GAGGCTTAGTGATGGTGCCT
Tnp1	ATGTCGACCAGCCGCAAGC	TCACAAGTGGGATCGGTAAT
Tnp2	TCGACACTCACCTGCAAGAC	ATCCTGGAGTGCGTCACTTG
Odf1	CCGCACTGAGTTGTCTTTTGG	GGGTGCATGTATAAGTCACACA
Akap4	GTCAGAAGGCGAGTTAAATCTGG	ATCCCTCCGTCTTAGACTGGT
Ropn1	CTGATCATCCACGCAGAT	ACTTCACACACTATCTTGAG
Prm1	GCCCACAAAATTCCACCTGC	CAAGATGTGGCGAGATGCTC
Cabs1	TGTGAACACCACAGATTTGCC	GAAGAGACCGGAGTTGGTGG
pre-*Tnp1*	ATGTCGACCAGCCGCAAGC	TGTTCCCCTGCTTGCTCAC
pre-*Tnp2*	TCGACACTCACCTGCAAGAC	TTGCAGTGTTCCCTTGTCCC
pre-*Prm2*	ACAAGACCATGAACGCGAGG	AGATGGGCAGCAGCAGCA
pre-*Odf1*	CCGCACTGAGTTGTCTTTTGG	AGGTCACACCCTCTCCCTTG
pre-*Akap4*	GTCAGAAGGCGAGTTAAATCTGG	TTTCCTTACCATCTCTGAACCAGAG
pre-*Ropn1*	CTGATCATCCACGCAGAT	CTCTGTCTACCTCGAGCCA
pre-*Prm1*	GCCCACAAAATTCCACCTGC	AGACTGCCCAGTCCCGCACC
pre-*Cabs1*	TGTGAACACCACAGATTTGCC	ACCCCTCGTGTATGTCTATGTGG

Atr	GAATGGGTGAACAATACTGCTGG	TTTGGTAGCATACACTGGCGA
BC026590	AAAGTGGCAAAGCCATTCAAAG	TCCATCAATCGACCTCTAACACA
Gfpt1	GAAGCCAACGCCTGCAAAATC	CCAACGGGTATGAGCTATTCC
Mdc1	GTGGCTCCTTGGGGTATAGTG	GGGCTTCGACCAACTACATTC
Ppp1cb	GATGTCGTCCAGGAAAGATTGT	TCAGTGGTGCTTCCAATTCCA
Psma8	GACAGGGCAATCACCGTCTTC	CGAGAGCGCAGATTTTCCTCA
Tox4	TCCCGGAGGAAATGACAATTACC	GTGAGGGATCAGAGTCCAAGG

Table S3. piRNA specific primers used in qRT-PCR

piRNA	Specific forward primer (5'-3')
piR-mmu-1	TGACATGAACACAGGTGCTCAGATAGCTTT
piR-mmu-17	TGCAAGGTGTCTTATGGGATTTGAAGTGT
piR-mmu-39	TGCAAGTGCGCTGACTTCCATTGGCACGAT
piR-mmu-59	TGCAAGTTGCTGGCTGTCGGATCTTAAA
piR-mmu-232	AAATGTACAGTGGTGACTACATACTTGC
piR-mmu-1937865	TATTGTTCTATGTTTTGTTGACTTGCGTTT
piR-mmu-1933172	TATCTGAAAAGGCTAGGAGGGCTAATCAG
piR-mmu-1930853	TATAGATACTGTTGCTTAATACTGAGCAC
piR-mmu-1928652	TATAAAAAGATATGTTTGCTTACCCTGA
piR-mmu-1927772	TAGTGCCTATGGGGTCTTACACAGAAAG

Table S4. Antibodies used in this study

Antibodies	Brand	Cat No.	RRID
anti-Ck137956	Abclonal	custom-made	-
anti-PIWIL1	Abcam	ab12337	AB_470241
anti-ACTIN	Abways	AB0033	-
anti-GAPDH	Abways	AB0036	-
anti-H4	Abcam	ab31830	AB_1209246
anti-HA	Sigma	H6908	AB_260070
anti-DDDDK-Tag	MBL	PM020	AB_591224
anti-β-TUBULIN	Abclonal	AC021	AB_2773004
anti-RPS3	Proteintech	11990-1-AP	AB_2180758
anti-MSY2	Proteintech	13538-1-AP	AB_2241777
anti-SYCP3	Proteintech	23024-1-AP	AB_11232426
anti-γH2AX	Abcam	ab26350	AB_470861
anti-MVH	Proteintech	51042-1-AP	AB_2092998
anti-LINE-1 ORF1p	Novus Biologicals	NBP2-66934	-
Anti-HDAC1	Santa Cruz	sc-81598	AB_2118083
Peanut Agglutinin (PNA), Rhodamine	VECTORLABS	RL-1072	AB_2336642
Donkey Anti-Mouse IgG H&L (Alexa Fluor® 555)	Abcam	ab150106	AB_2857373
Goat Anti-Rabbit IgG H&L (Alexa Fluor® 555)	Abcam	ab150078	AB_2722519
Goat Anti-Mouse IgG H&L (Alexa Fluor® 488)	Abcam	ab150113	AB_2576208
Goat Anti-Rabbit IgG H&L (Alexa Fluor® 488)	Abcam	ab150077	AB_2630356
Goat anti-Mouse IgG (H+L) Secondary Antibody, HRP	Invitrogen	31430	AB_228307
Goat anti-Rabbit IgG (H+L) Secondary Antibody, HRP	Invitrogen	65-6120	AB_2533967
