# Supplementary material for: Regulation of Miwi-mediated mRNA stabilization by Ck137956/Tssa is essential for male fertility
Source: BMC Biol. 2023 Apr 17;21:89. doi: 10.1186/s12915-023-01589-z (PMC10111675; doi:10.1186/s12915-023-01589-z)

Fig 1A

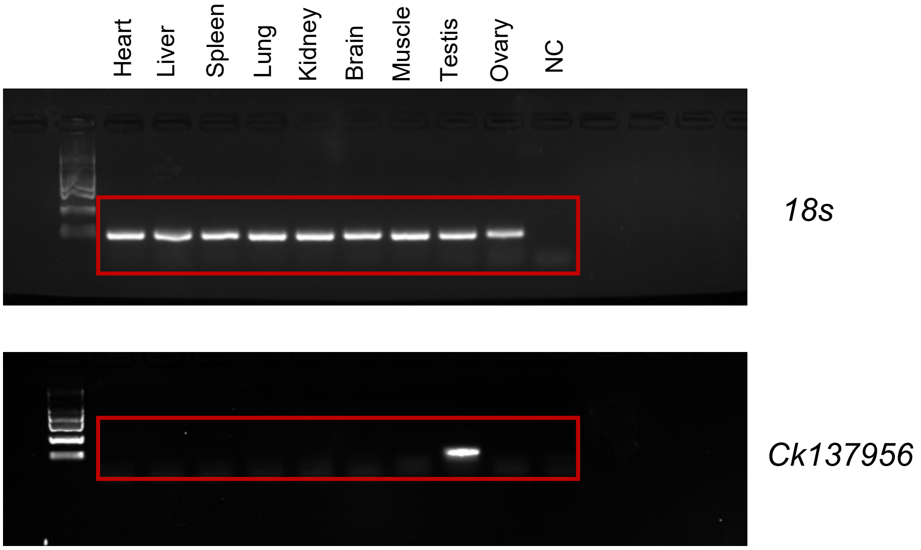

Heart
Liver
Spleen
Lung
Kidney
Brain
Muscle
Testis
Ovary
NC
18s
Ck137956

Fig 1C

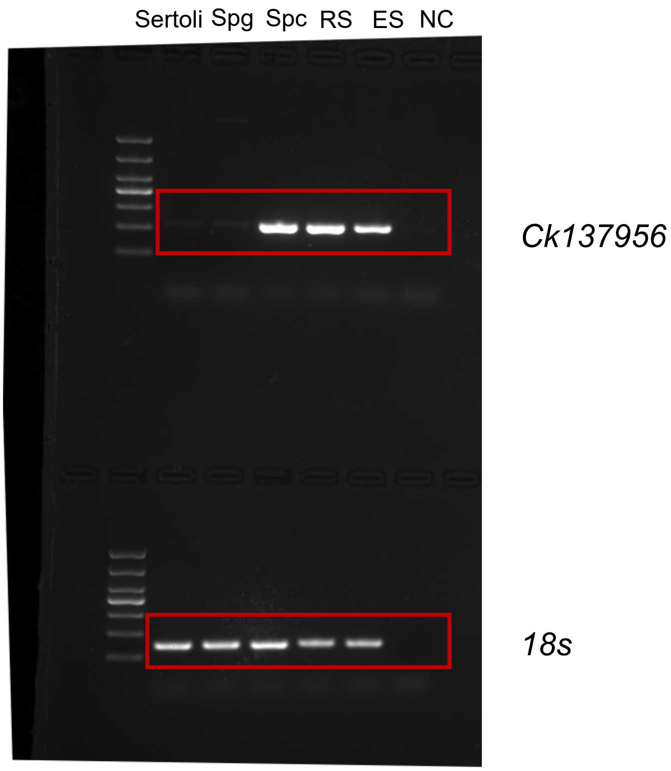

Fig 1H

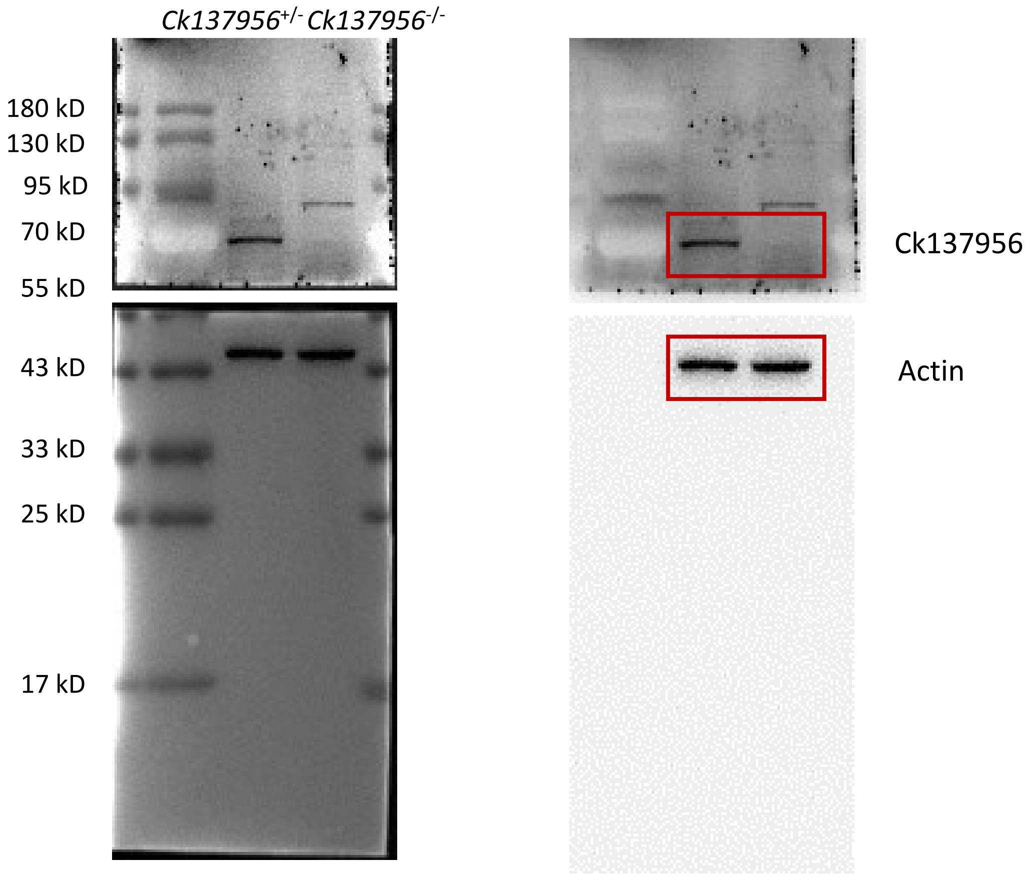

Ck137956+/- Ck137956-/-
180 kD
130 kD
95 kD
70 kD
55 kD
43 kD
33 kD
25 kD
17 kD
Ck137956
Actin

Fig 3G

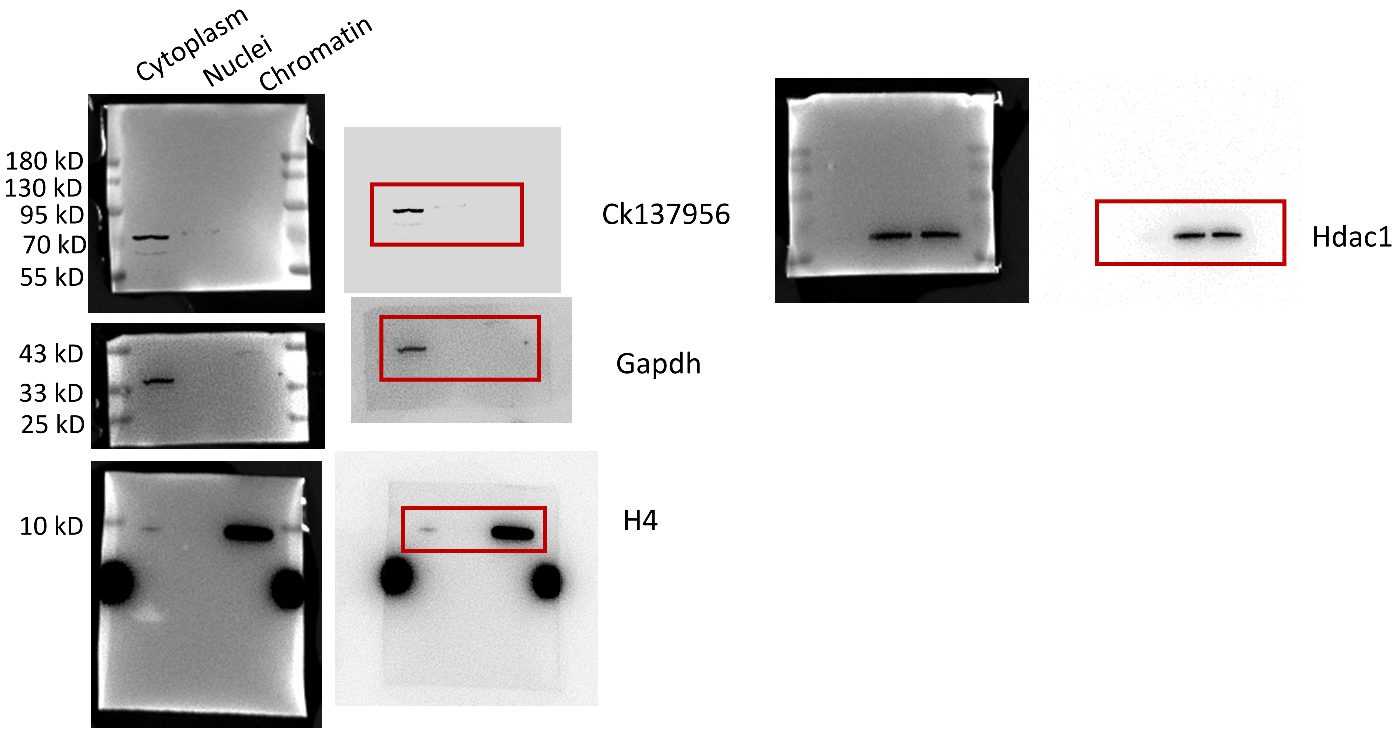

Cytoplasm
Nuclei
Chromatin
180 kD
130 kD
95 kD
70 kD
55 kD
Ck137956
43 kD
33 kD
25 kD
Gapdh
10 kD
H4
Hdac1

Fig 4A

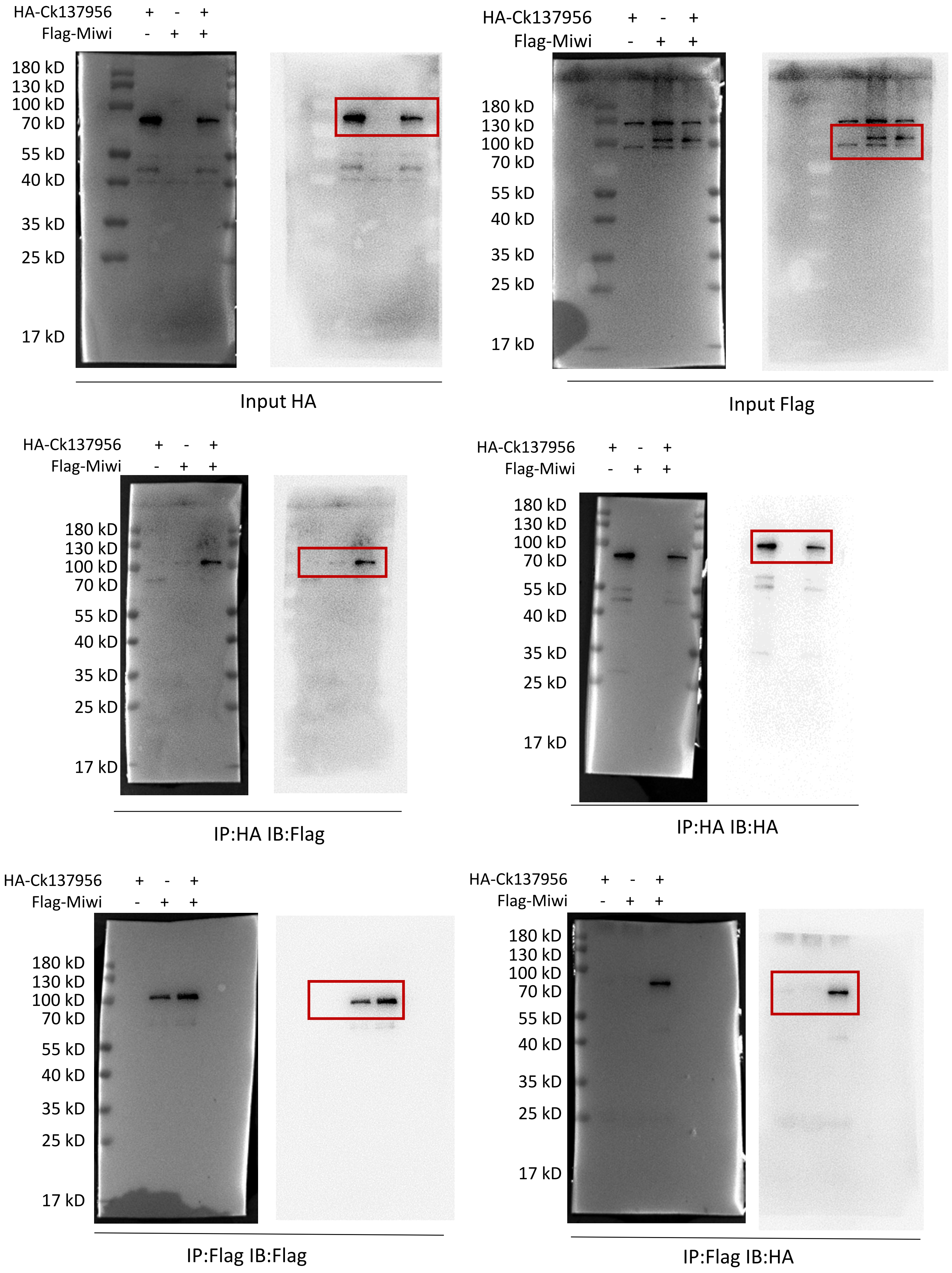
HA-Ck137956 + - +
Flag-Miwi - + +
180 kD
130 kD
100 kD
70 kD
55 kD
40 kD
35 kD
25 kD
17 kD
Input HA
Input Flag
IP:HA IB:Flag
IP:HA IB:HA
IP:Flag IB:Flag
IP:Flag IB:HA

Fig 4B

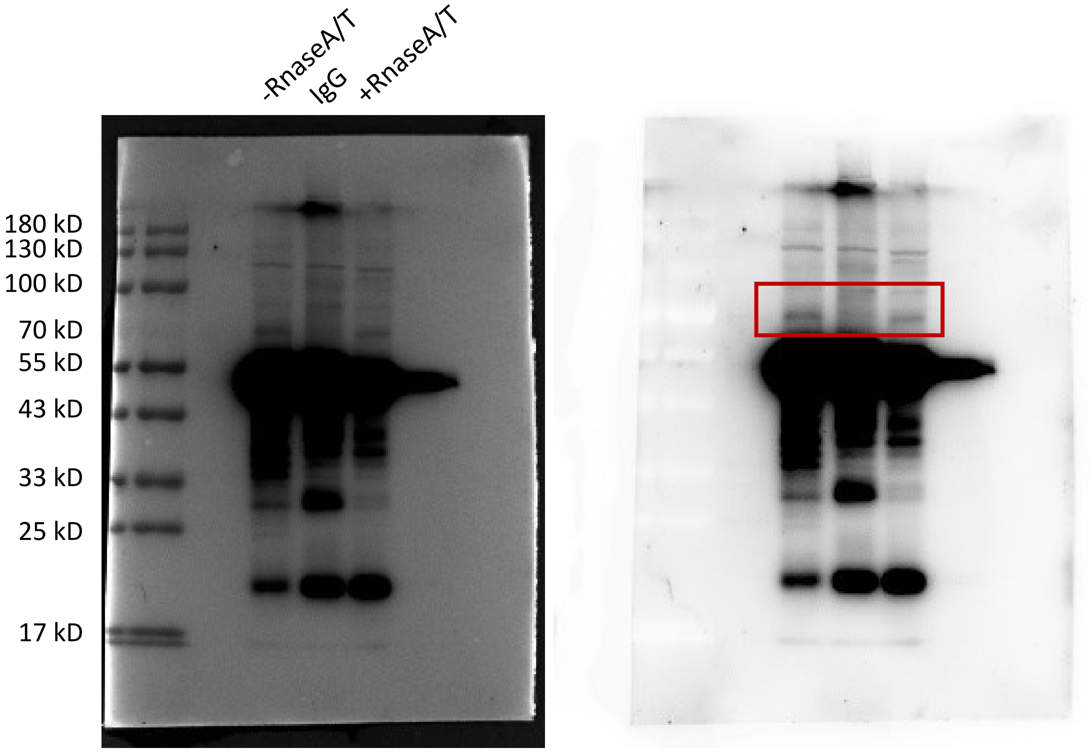

IP: Miwi IB: Ck137956

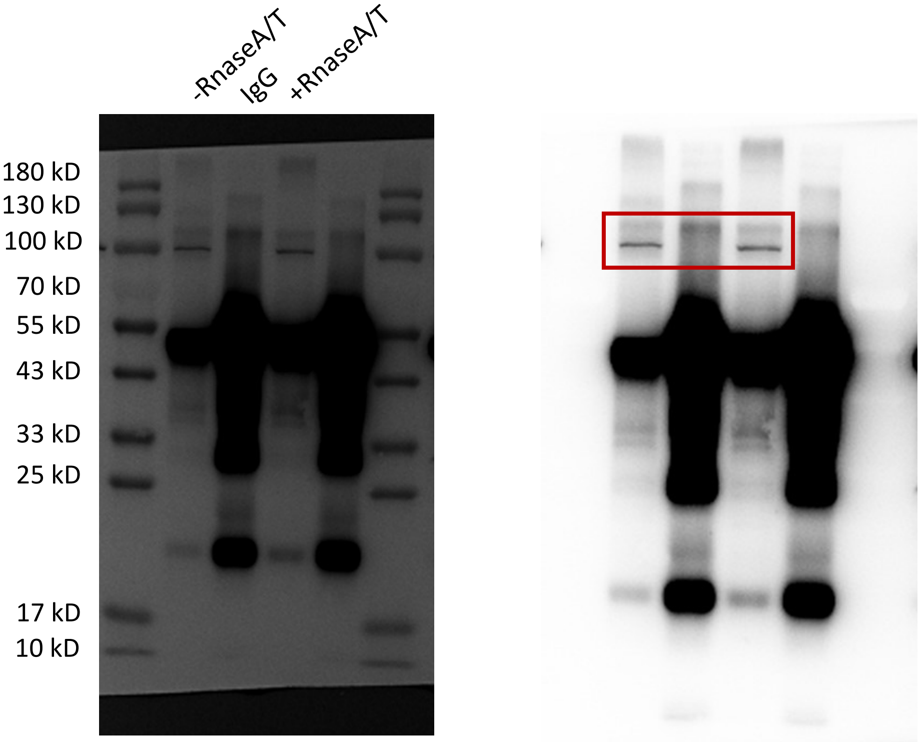

IP: Ck137956 IB: Miwi

Fig 4C

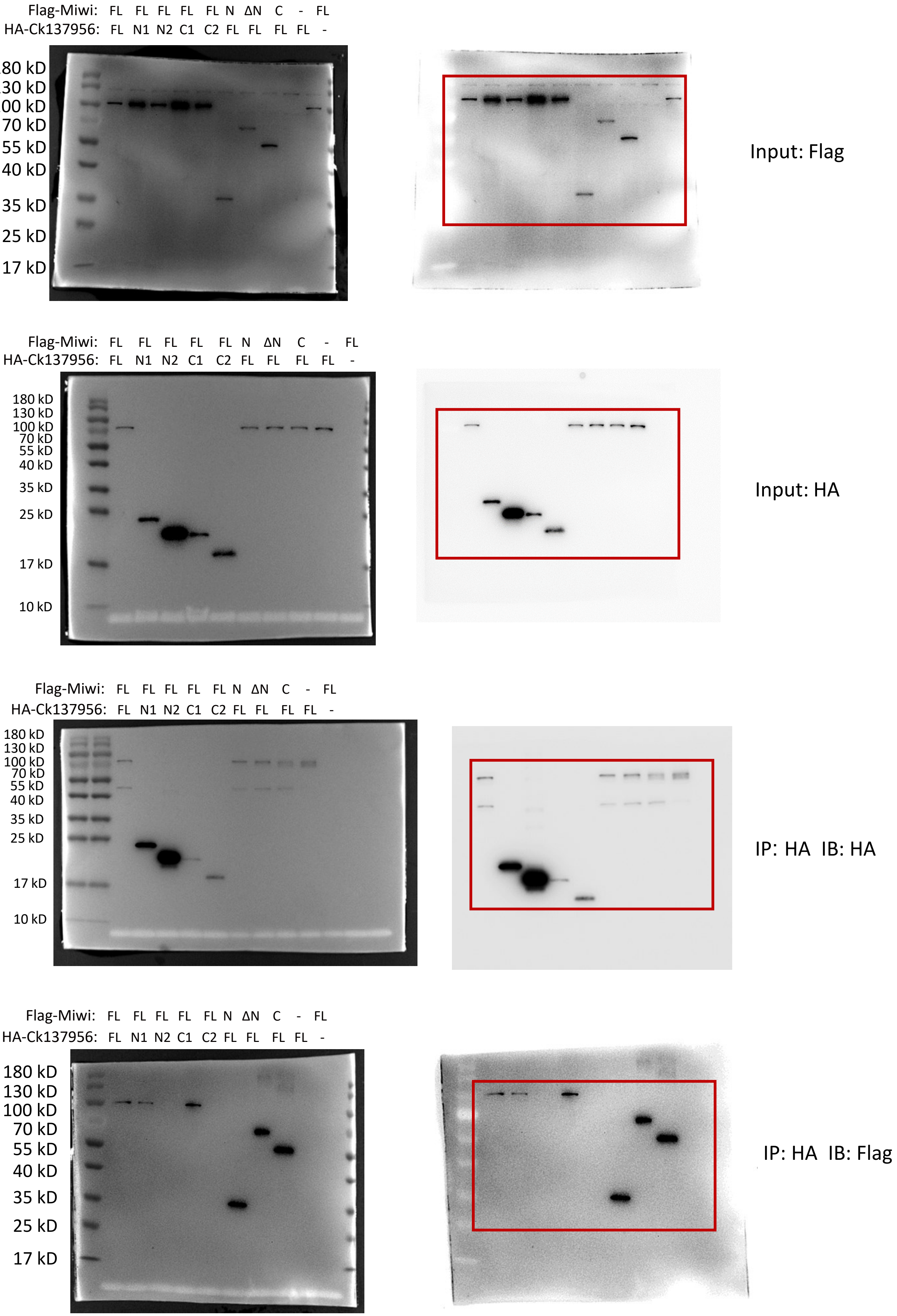
Flag-Miwi: FL FL FL FL FL N ΔN C - FL
HA-Ck137956: FL N1 N2 C1 C2 FL FL FL FL -
180 kD
130 kD
100 kD
70 kD
55 kD
40 kD
35 kD
25 kD
17 kD
Input: Flag
Flag-Miwi: FL FL FL FL FL N ΔN C - FL
HA-Ck137956: FL N1 N2 C1 C2 FL FL FL FL -
180 kD
130 kD
100 kD
70 kD
55 kD
40 kD
35 kD
25 kD
17 kD
10 kD
Input: HA
Flag-Miwi: FL FL FL FL FL N ΔN C - FL
HA-Ck137956: FL N1 N2 C1 C2 FL FL FL FL -
180 kD
130 kD
100 kD
70 kD
55 kD
40 kD
35 kD
25 kD
17 kD
10 kD
IP: HA IB: HA
Flag-Miwi: FL FL FL FL FL N ΔN C - FL
HA-Ck137956: FL N1 N2 C1 C2 FL FL FL FL -
180 kD
130 kD
100 kD
70 kD
55 kD
40 kD
35 kD
25 kD
17 kD
IP: HA IB: Flag

Fig 4D

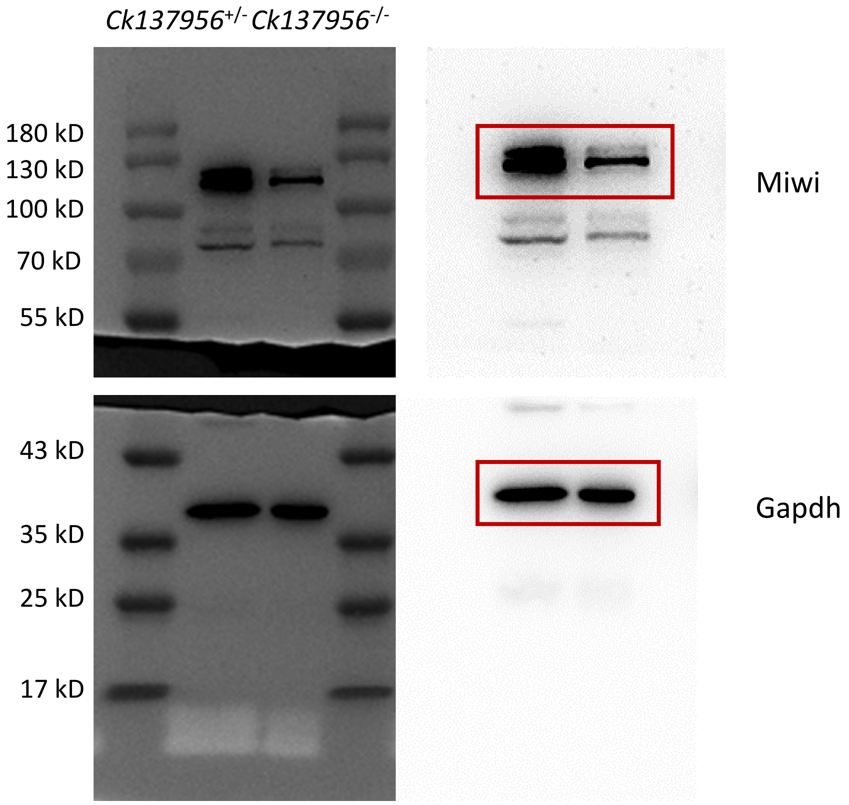
Ck137956+/- Ck137956-/-
180 kD
130 kD
100 kD
70 kD
55 kD
43 kD
35 kD
25 kD
17 kD
Miwi
Gapdh

Fig 4F

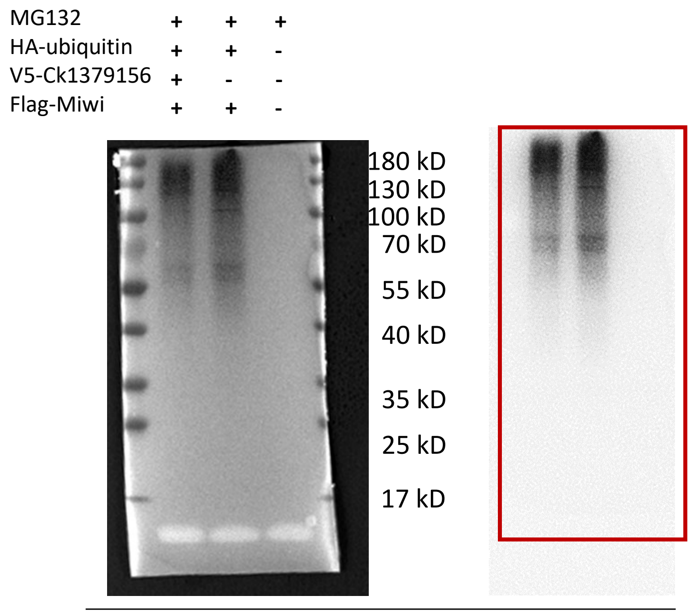

IP: Flag IB: HA

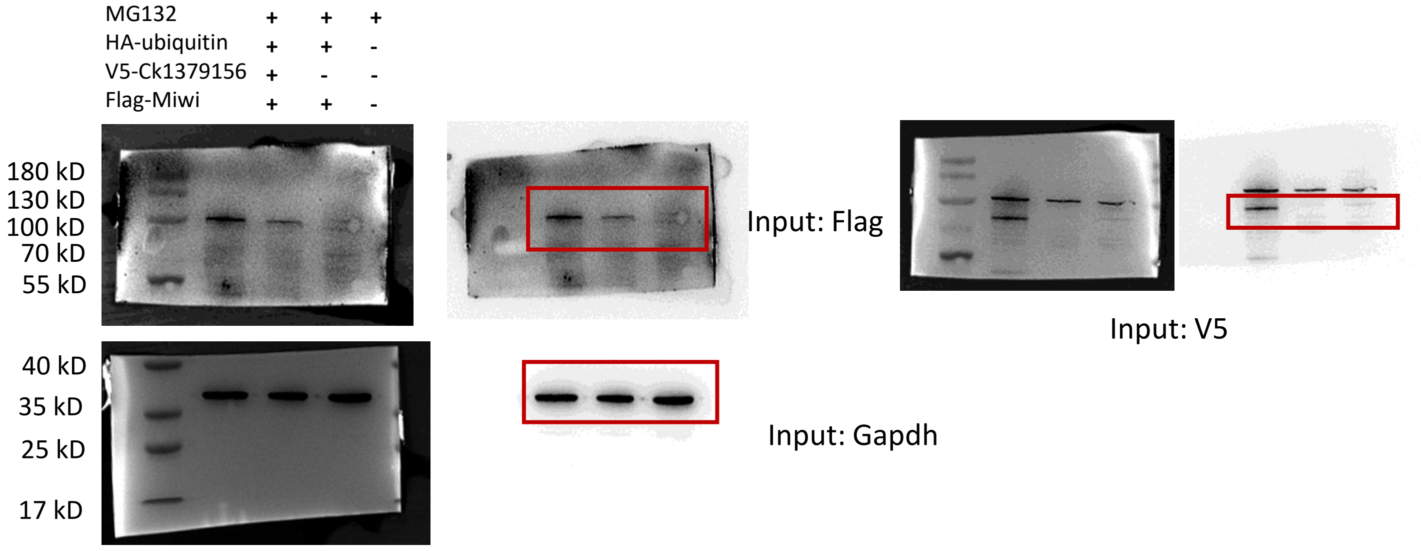

Fig 5C

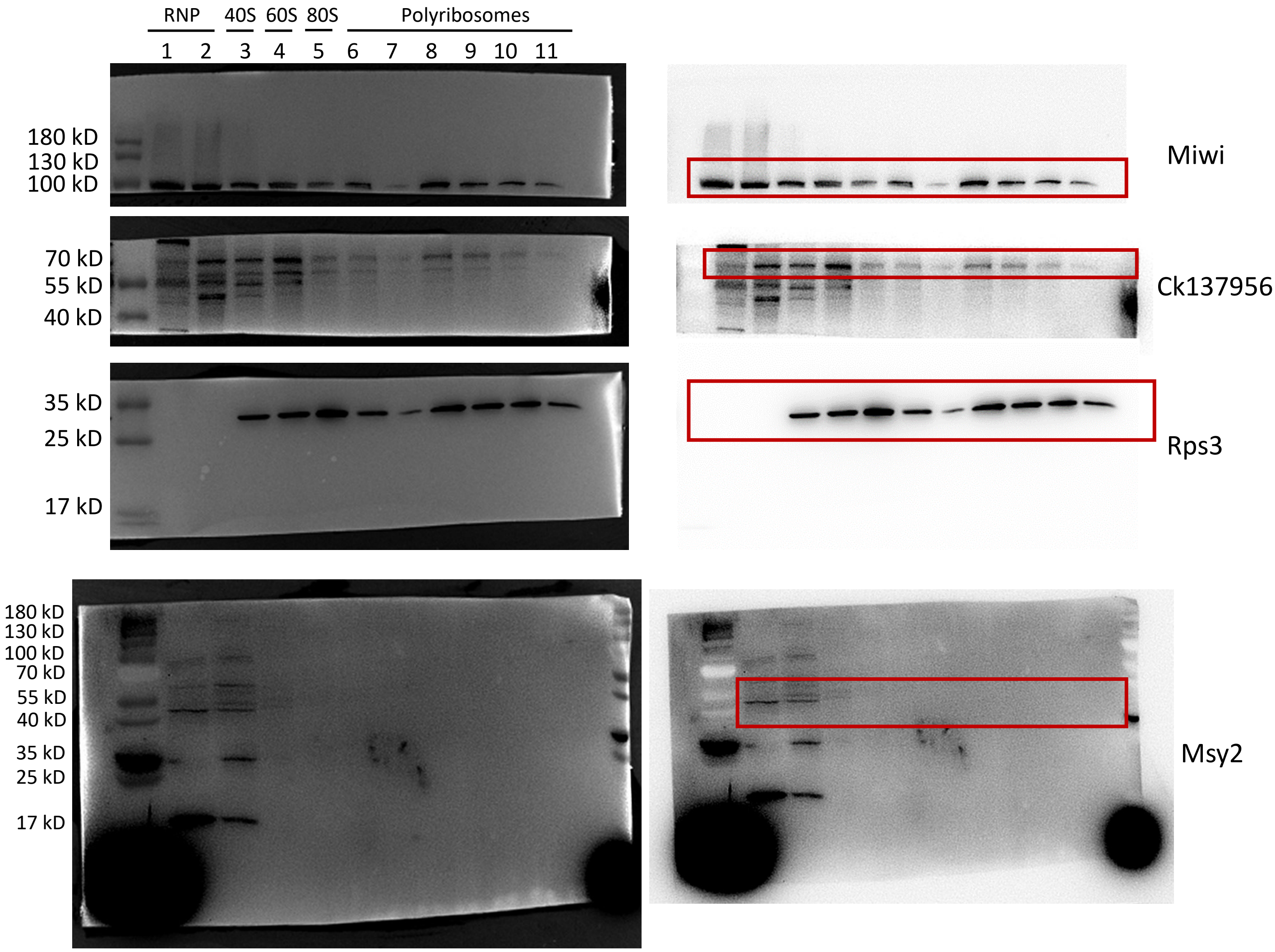
RNP
40S
60S
80S
Polyribosomes
1 2 3 4 5 6 7 8 9 10 11
180 kD
130 kD
100 kD
70 kD
55 kD
40 kD
35 kD
25 kD
17 kD
Miwi
Ck137956
Rps3
Msy2

Additional file 1: Fig S1A

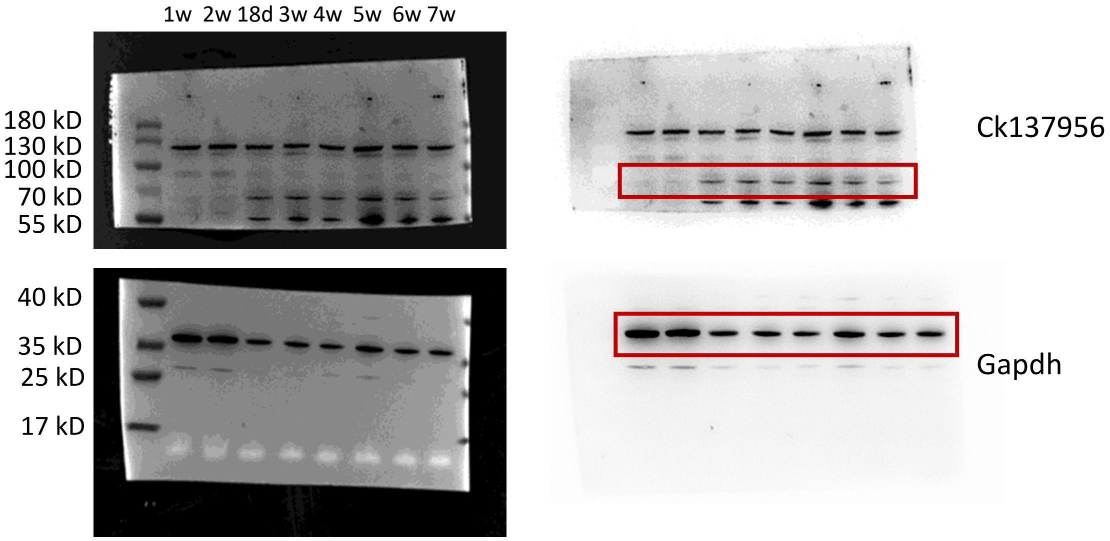

Additional file 1: Fig S3D

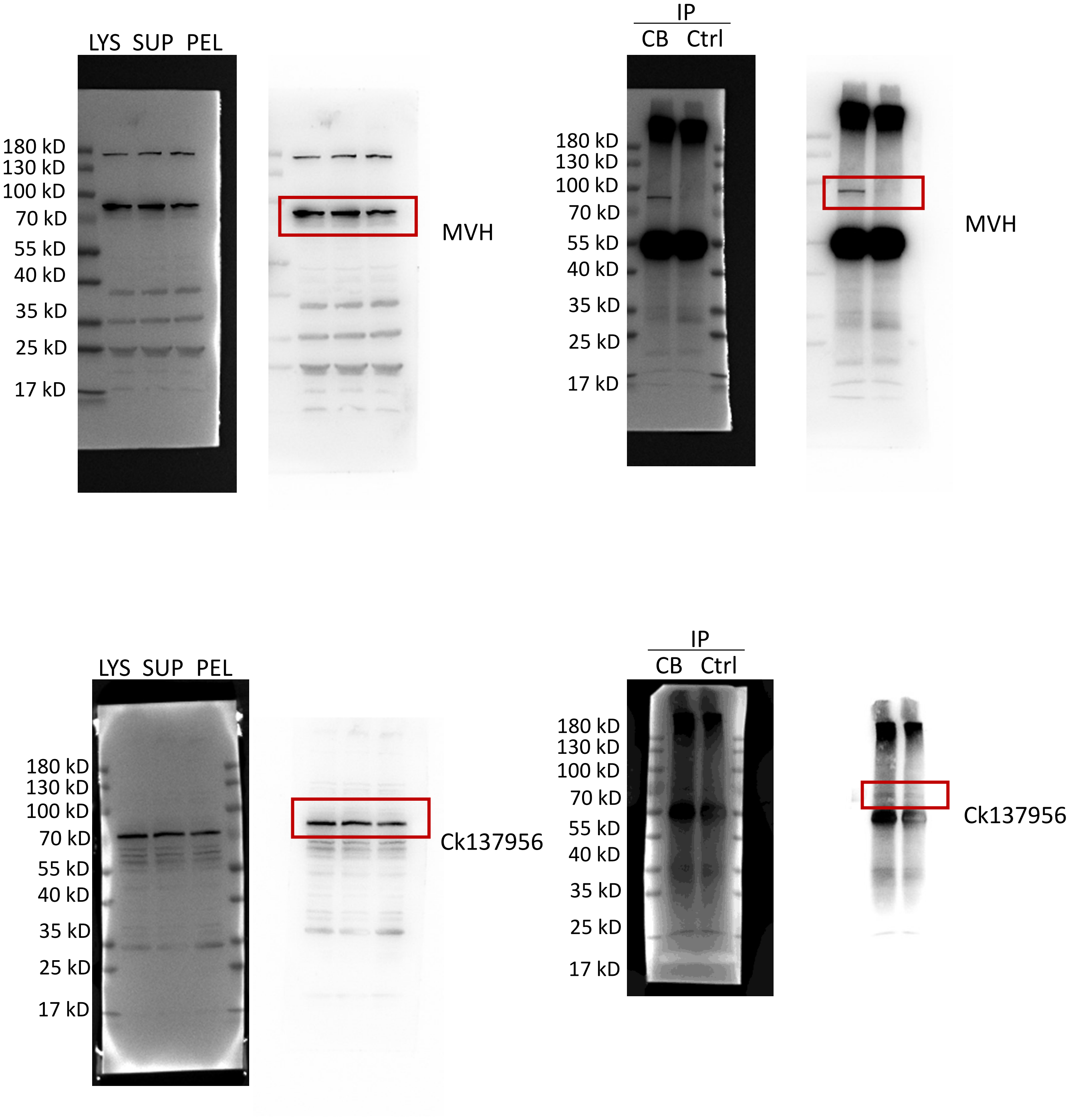

Additional file 1: Fig S4B

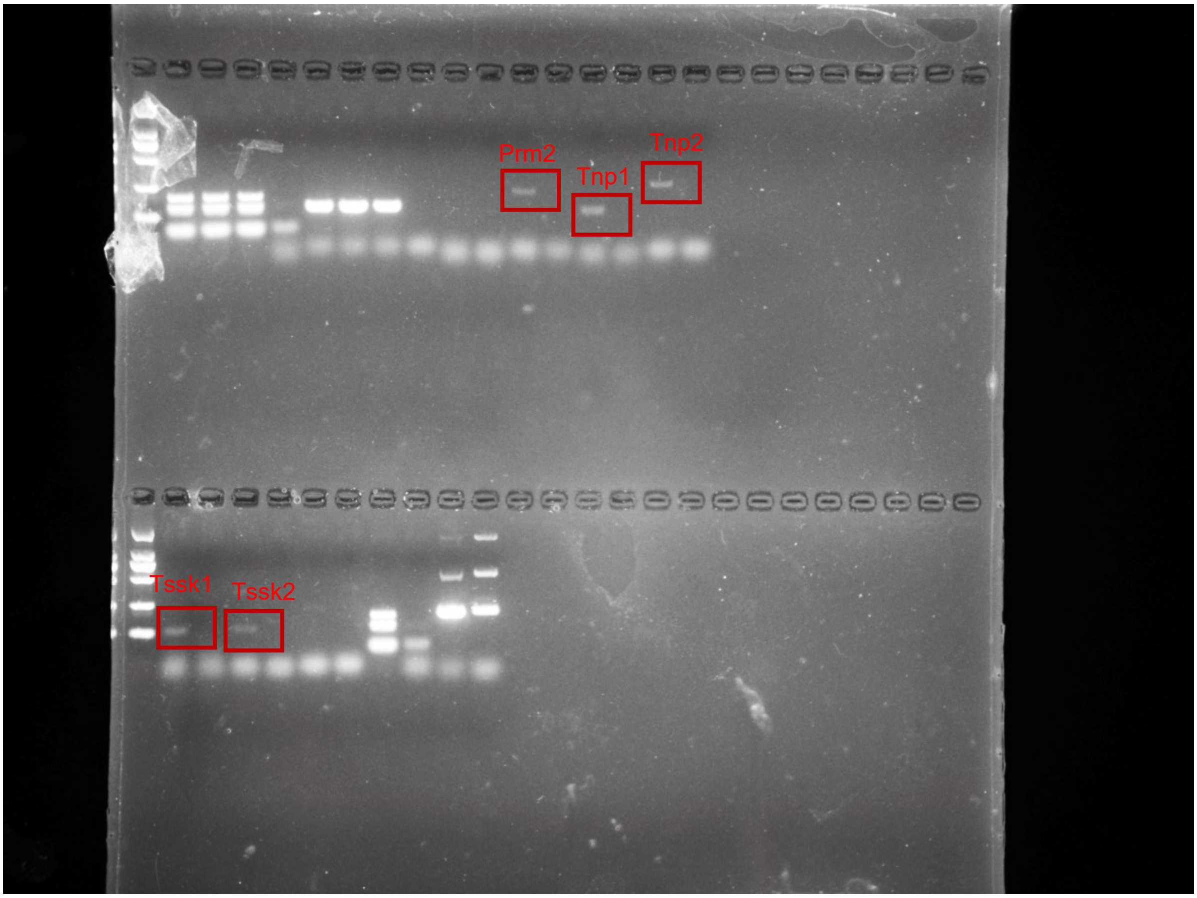

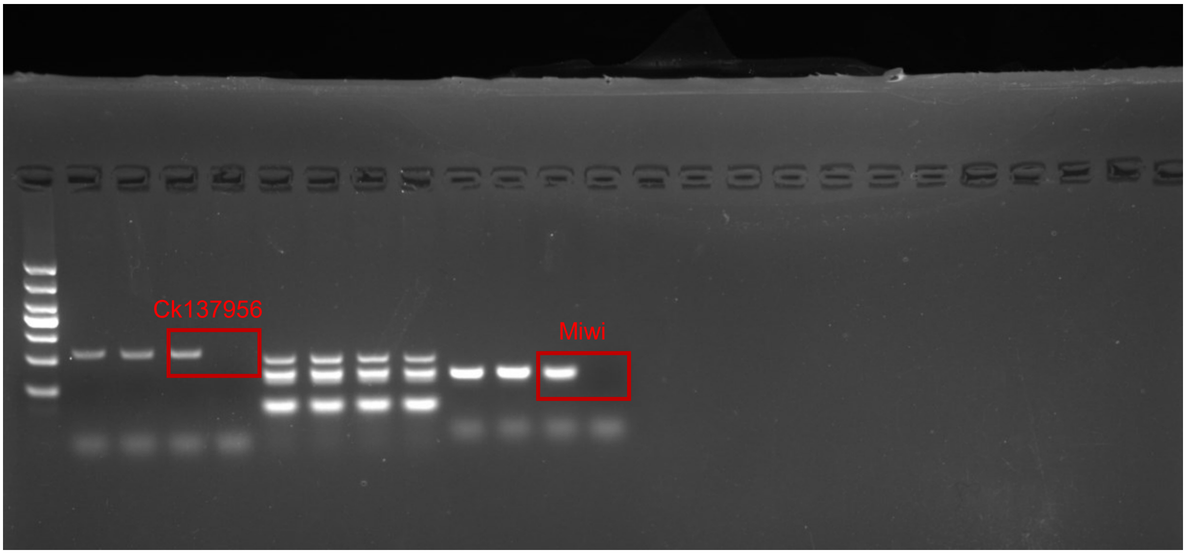

Supplement: Supplementary file 8 — Additional file 8. Uncropped gels/blots. [file 12915_2023_1589_MOESM8_ESM.pdf]
